# Ridge-assisted micro positioning of cells in a channel

**DOI:** 10.1126/sciadv.aec9227

**Published:** 2026-07-17

**Authors:** Adriana Payan-Medina, Victor R. Putaturo, Sajad R. Bazaz, Quinn E. Cunneely, Varun Kulkarni, Mehmet Toner, Avanish Mishra

**Affiliations:** ^1^ Harvard-MIT Division of Health Sciences and Technology, Cambridge, Massachusetts 02139, USA.; ^2^ Center for Engineering in Medicine and Surgery, Massachusetts General Hospital and Harvard Medical School, Charlestown, Massachusetts 02129, USA.; ^3^ Krantz Family Center for Cancer Research, Massachusetts General Hospital, Mass General Brigham Cancer Institute and Harvard Medical School, Charlestown, Massachusetts 02129, USA.; ^4^ Department of Surgery, Massachusetts General Hospital, Mass General Brigham and Harvard Medical School, Boston, Massachusetts 02114.; ^5^ School of Engineering and Applied Sciences and Wyss Institute for Biologically Inspired Engineering, Harvard University, Cambridge, MA 02138.; ^6^ Shriners Children’s, Boston, MA 02114, USA.

## Abstract

The ability to deterministically and precisely control the lateral position of focused cell streams within a microchannel enables a wide range of biomedical applications, including cell concentration, sorting, and sensing. By leveraging engineered microvortices generated by channel ridges and fluidic lift forces, we present a microfluidic method for deterministically positioning focused cell streams to desired streamlines. Unlike inertial focusing, ridge-assisted micro positioning (RAMP) is independent of particle size and flow rate, enabling polydisperse particles to be focused to the same streamline across a broad range of flow rates. The focusing position can be precisely adjusted by altering the ridge placement and geometry, allowing for micron-level lateral shifts in a controlled manner. Applying the RAMP concept, we demonstrate clog-free microfluidic cell concentrators that can enrich single cells and clusters to desired concentration factors and show the ability to focus particles in undiluted whole blood. Together, these results establish RAMP as a versatile platform for precise cell positioning in complex biological fluids.

## Teaser

Ridge-produced vortices allow cells to be focused in user-defined streamlines over a wide range of flow rates and particle sizes.

## INTRODUCTION

Microfluidic devices are invaluable tools for analysing complex biofluids, such as blood, pleural, and peritoneal fluids, for disease monitoring and diagnosis (*1*–*4*). These samples vary substantially in composition across patients and contain heterogeneous cell populations (*5*). For such analyses, precise cell positioning within a microchannel is critical. Flow cytometry, cell sorting, and sensing all rely on consistent placement of cells at streamlines where sorting forces or sensing fields are optimal, thereby improving measurement sensitivity and accuracy (*6*). Conventional flow cytometry systems achieve this two-dimensional alignment using coflowing buffer sheath streams to confine cells at the centre of the optical path (*7*). Sheath streams have also been used to tightly order cells in all three dimensions in imaging flow cytometry to reduce out-of-focus dropout events (*8*, *9*).

**Fig. 1.**
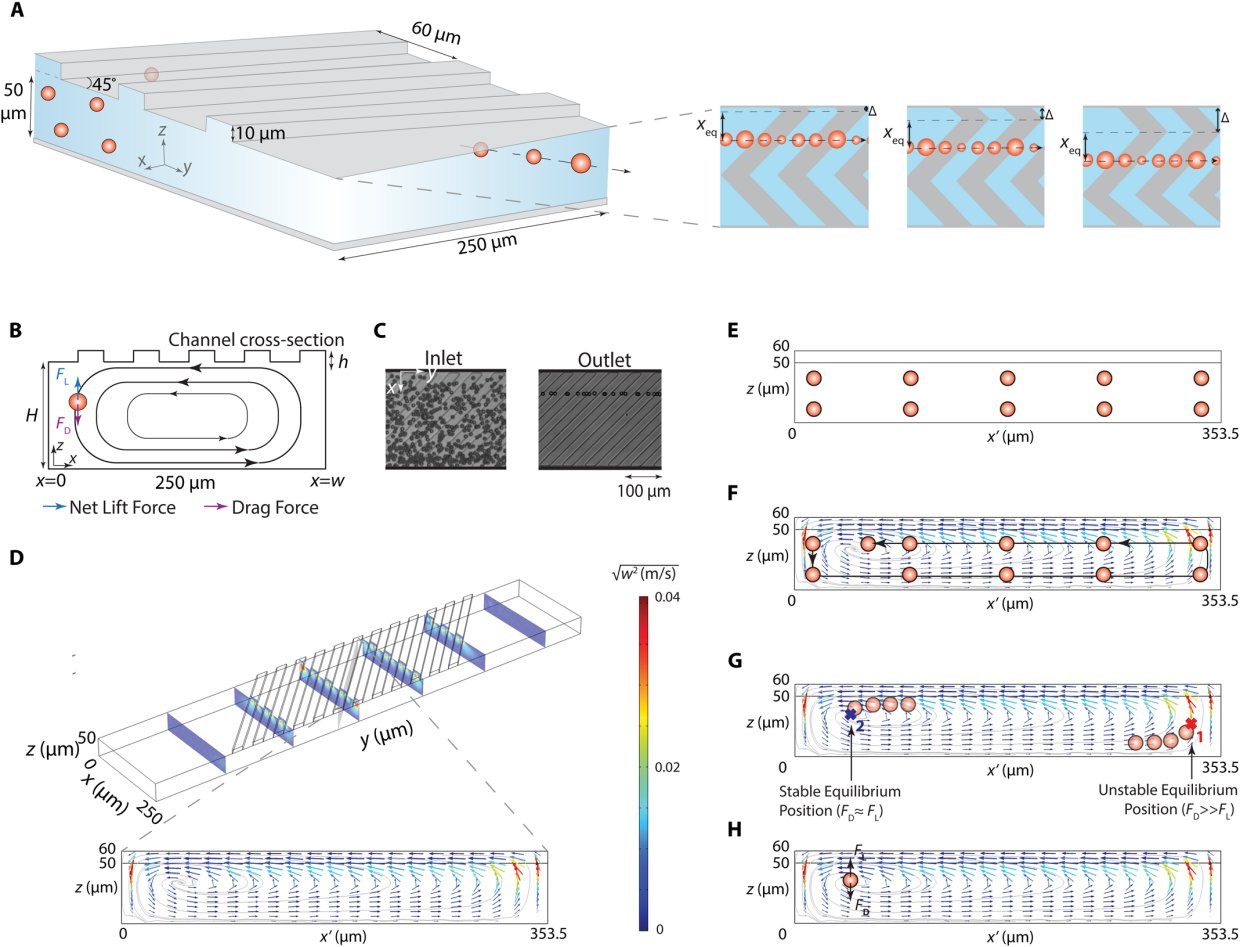
Ridge-assisted micro positioning (RAMP) achieves controllable focusing of polydisperse particle streams. (**A**) A schematic representation of a RAMP device shows the inertial focusing effect observed in a microchannel due to a transverse vortex caused by ridges. Channels with RAMP can precisely adjust the focusing position of particles as the ridge structure location is shifted (ridge shift indicated with Δ, while the particle focusing position, *x_eq_*, remains constant). Shifts in central ridge position (Δ) result in proportional, micron-level shifts in particle focus position. (**B**) The microvortex exerts a drag force on particles. When drag and lift forces are balanced, particles assume a stable equilibrium position. (**C**) High-speed microscopy images show dispersed 10 μm particles at the inlet and focused particles at the outlet at 500 μl/min (Re = 50.4). (**D**) Finite element modelling results (fig. S1) demonstrate transverse velocity vectors and flow recirculation regions, color-mapped by the *z*-component of velocity (∣w∣), at a cross-section parallel to the ridge microstructure (fig. S2). (**E** to **H**) Mechanism of particle focusing. In a rectangular channel without ridges, particles undergo inertial ordering along the long faces of the channel (E). Transverse vortex, produced by ridges, sweeps particles (F). Theoretically, two equilibrium positions are possible (G), one near the center of the vortex (2) and another near a sidewall, away from the center of vortex (1), but drag force *F_D_* is stronger near position-1, causing particles to be swept from position-1 to a stable equilibrium position-2, near the stagnation region at the center of the vortex where *F_D_* is feeble (H).

Inertial microfluidics has provided elegant approaches for high-throughput cell ordering (*10*–*16*) (table S1). However, the relative focusing position is predetermined by the channel geometry and cannot be independently controlled. Thus, a key unmet need has been the development of a microfluidic system that achieves deterministic focusing and permits precise (micron-level) adjustable control of lateral cell positioning. In this study, we introduce a ridge-assisted micro positioning (RAMP) platform that enables polydisperse particles to be focused into a single streamline. Critically, the lateral position of the focused stream can be predictably adjusted by modifying the ridge placement and geometry, enabling micron-level control from the channel sidewall to its centreline (Fig. 1A). This tunability distinguishes RAMP from previous inertial focusing approaches and establishes a versatile foundation for a range of biomedical applications.

## RESULTS

### RAMP design and focusing principles

The focusing mechanism for RAMP can be attributed to secondary flows generated by the ridges incorporated into the microchannel (Fig. 1, A to D). In a rectangular channel without microstructures, particles migrate to equilibrium positions near the long face of the channel, where shear gradient and wall-induced lift forces oppose one another (Fig. 1E) (*17*, *18*). The introduction of ridges disrupts this balance by inducing anisotropic pressure gradients along their inclined surfaces, which drive fluid recirculation parallel to the ridge direction (Fig. 1B) (*19*). This recirculating flow forms a transverse vortex that exerts a drag force on suspended particles. In combination with inertial lift forces, this drag force guides particles toward a stable focusing position (Fig. 1, C and D) (*17*).

The approximate lift and drag force equations for particle trapping are described in the Supplementary Text with eqs. S1 to S3. A simplified ratio of these two forces (*F_L_/F_D_*) (eq. S3) demonstrates that particle focusing is dictated by the particle diameter, *a*, confinement ratio, *a/D_h_*, where *D_h_* is the hydraulic diameter of the channel, and mean flow velocity, *U*. The fluid speed along the ridges, which can act as an indicator of the secondary flow, is a quadratic function of the geometrical non-dimensional factor, α=h/2H′, where *h* is the ridge height and H′=H+h/2 (eq. S4) (*20*). The strength of the transverse flow can be further tuned with the ridge geometry (angle, θ, height, *h*, and wavelength, λ), channel dimensions (width, *W* and height, *H*), and Reynolds number (Re) (*19*).

Based on this analysis, the ridge and channel dimensions were designed to have a low α (∼0.1) and a high confinement ratio (≥0.1), allowing for a balance of lift-to-drag forces ($F_{L}\approx F_{D}$) (Fig. 1). The resulting focusing effect shows 10 μm particles align near the channel sidewall at the outlet, contrasting with the dispersion observed at the inlet (Fig. 1C). In this case, microchannels are 250 μm wide (*W*, *x*-direction), 50 μm deep (*H*, *z*-direction), and Re is 50.4 while the particle confinement ratio is 0.12. The ridges are 10 μm high, with a 60 μm wavelength. Ridges are angled at 45° with respect to the long axis of the channel (Fig. 1A).

Finite element modeling of fluid flow (fig. S1) demonstrates the ridge-induced microvortex (Fig. 1F) in a cross-section parallel to the ridge (fig. S2). In a low-aspect-ratio rectangular channel with ridge-induced vortices, at moderate to high Re (5 to 250), theoretically two focusing positions, 1 and 2, are possible (Fig. 1G), each near the channel sidewall. The key difference between positions 1 and 2 is a higher vertical component of velocity at position-1, as shown in Fig. 1, F to H. Here, velocity vectors are color-mapped to the *z*-component of velocity (∣w∣). Near the distant sidewall (position-1), the *F_L_/F_D_* ratio varies from 0.01 to 0.23, indicating an unstable equilibrium position. This ratio of forces was calculated using eq. S3 for a flow rate of 500 μl/min (Re = 50.4). Lift coefficient, *C_L_* was estimated from the previously published work (*21*, *22*), and *w* was computationally deduced at various *z*-positions. This is an approximate analysis based on an empirical equation of lift force and the velocity, *w*, estimated using computational modeling, but it provides important insights into focusing behavior. Thus, particles at the unstable equilibrium position-1 are swept away to a stable equilibrium position-2, near the center of the vortex, where the transverse component of the velocity (*w*) is negligible, allowing balancing of the lift and drag forces (*F_L_ = F_D_*) (Fig. 1H). This focusing behavior is experimentally demonstrated in fig. S1D, which shows a vortex sweeping green-fluorescent 10 μm particles to position-2. This is further supported by fluid flow simulations showing the stagnation region at the center of the vortex at *z* = 34 μm, concordant with experimentally observed focusing at *z* = 31.5 ± 0.6 μm. Thus, the stagnation zone at the center of the vortex indicates the stable trapping region.

### RAMP for precise adjustment of particle focus position

**Fig. 2.**
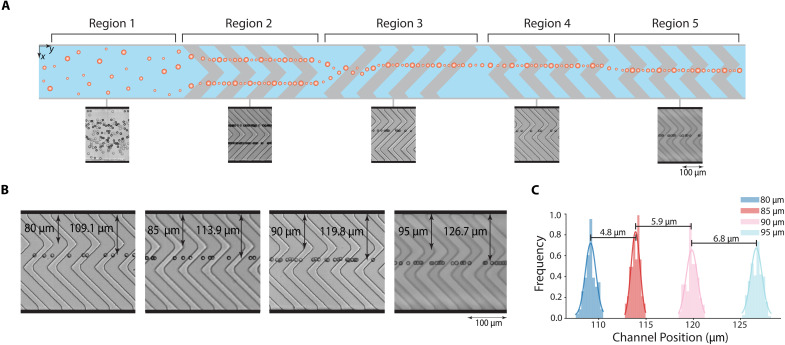
The RAMP device can achieve shifts in particle focus position through proportional shifts in ridge position. (**A**) Particles at the inlet (region 1) flow into symmetrical ridges in region 2 that align particles into two streamlines. Region 3 uses a long, single ridge to align particles into a single line near the channel center. In region 4, particles flow into a region with an alternative central ridge structure. Between regions 4 and 5, the central ridge location is shifted, resulting in proportional shifts in the particle focus position. (**B**) High-speed streak images with reference distances reveal that the focused particle stream shifts proportionally with the central ridge position as it is shifted from 80 μm [image reused from panel (A), region 4] to 95 μm [image reused from panel (A), region 5] from the channel sidewall. (**C**) A frequency distribution of particle centroid positions at ridge shifts of 80 μm (*n* = 83), 85 μm (*n* = 65), 90 μm (*n* = 58), and 95 μm (*n* = 314).

First, we show that RAMP provides precise control of particle positioning across the channel cross-section. Shifting the position of the ridge structure within the channel results in proportional shifts in the particle focus position (Fig. 2A, regions 4 and 5). To demonstrate this capability, we microfabricated two sets of RAMP devices designed for either small (5 μm) or large (15 μm and 30 μm) focus-position shifts. In these devices, microparticles are first aligned on both sides of a chevron microstructure within the channel, arranging particles into two streamlines (Fig. 2A, region 2). Following the chevron region, a region with a central ridge structure joins these streamlines into a single streamline near the channel center (Fig. 2A, region 3). Once particles are concentrated in a common region within the channel, small or large shifts in focus position are created by shifting the central ridge (Fig. 2A, regions 4 and 5, and fig. S3A).

Shifts in the location of the central ridge create a proportional shift in the particle focus position. As the central ridge structure is shifted in steps of 5 μm from 80 μm to 95 μm in the mylar mask design (Fig. 2B), secondary flow created by this structure caused focused particle streams to shift in steps of 4.8 ± 0.7 μm, 5.9 ± 0.8 μm, and 6.8 ± 0.9 μm, respectively (Fig. 2, B and C, and table S2). Slight variations between the targeted 5 μm shift in the design to experimentally achieved shifts can be attributed to SU-8 photolithography tolerances, where features from the digital design are not exactly reproduced in SU-8, especially in this case, where a mylar mask was used, which has slightly lower resolution than glass chrome masks. A high-speed imaging video of 10 μm particles flowing through devices with central ridge structures at 80 μm and 90 μm further illustrates this controlled shift (movie S1). Similarly, as a central ridge structure is shifted in larger steps of 15 μm and 30 μm, proportionate focus position shifts of 17.1 ± 0.9 μm and 29.6 ± 1.2 μm are achieved (fig. S3, B and C, and table S2). To demonstrate that this concept also works with polydisperse particles, we tested a mixed population of 10 μm and 15 μm particles flowing through the RAMP device (fig. S4). As expected, 5 μm shifts in ridge location from 80 μm to 90 μm resulted in particle focus position shifts of 5.7 ± 1.3 μm and 6.4 ± 1.3 μm, respectively (fig. S4B and table S2), demonstrating the remarkable lateral positioning capability of this approach for particles of various sizes. Again, slight deviations from the targeted design steps of 5 μm can be attributed to SU-8 microfabrication tolerances. These results confirm that RAMP enables deterministic and finely tunable lateral positioning of monodisperse and polydisperse particle populations.

### RAMP facilitates focusing of polydisperse particles across a wide flow rate range

**Fig. 3.**
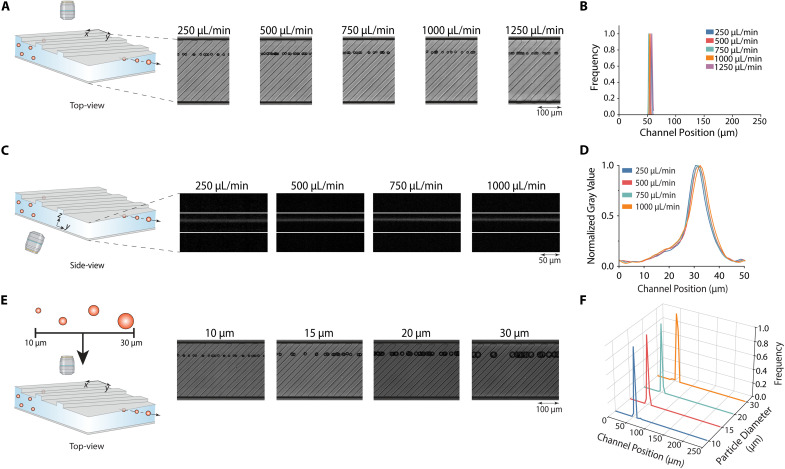
RAMP device with straight ridges enables focusing of particles across a wide range of flow rates in *x*, *y*, and *z* directions with particles of different sizes. (**A**) High-speed streak imaging shows that 10 μm particles maintain their focus position at various flow rates. (**B**) A frequency distribution diagram of particle centroid positions quantifies dispersion in focusing position at flow rates of 250 μl/min (*n* = 96, *Re* = 25.2), 500 μl/min (*n* = 166, *Re* = 50.4), 750 μl/min (*n* = 111, *Re* = 75.6), 1000 μl/min (*n* = 114, *Re* = 100.8), and 1250 μl/min (*n* = 110, *Re* = 126.0). (**C**) Fluorescent microscopy images show a side-view of 10 μm particles in the *y-z* plane. (**D**) The fluorescent intensity profile of the focused streamline shows that particles focus in the same *z*-position for flow rates ranging from 250 to 1000 μl/min (*n* = 20 profiles per flow rate). (**E**) High-speed streak imaging provides a top view of particles with diameters ranging from 10 to 30 μm, all aligned at nearly the same position in the channel. The image for 10 μm particles is repurposed from Fig. 1. (**F**) Frequency distributions of particle centroid positions for 10 μm (*n* = 104), 15 μm (*n* = 100), 20 μm (*n* = 69), and 30 μm (*n* = 102) particles, showing focus position data for a flow rate of 250 μl/min. Data for other flow rates follow a similar trend (fig. S9). Created in BioRender. Payan-medina, A. (2026) https://BioRender.com/i4yx21j.

We next examined whether RAMP could maintain stable polydisperse particle focusing across a broad flow rate regime. Fig. 3A shows streak images of 10 μm particles at flow rates ranging from 250 to 1250 μl/min (*Re* = 25.2 to 126.0). In a 250 μm-wide cross-section, transverse to the flow, 10 μm particles focused between 52.2 ± 0.5 and 57.8 ± 0.7 μm (table S3). A frequency distribution plot of the particle focusing position illustrates that the equilibrium position remains consistent across this flow rate range, with negligible dispersion (Fig. 3B). To define the operating regime of RAMP, flow rates ranging from 10 to 2500 μl/min (*Re* of 1 to 252) were tested (fig. S5). 10 μm particles focus over a flow rate range of 100 to 2500 μl/min (*Re* = 10.1 to 252.0) while 20 μm particles focus over a flow rate range of 50 to 2500 μl/min (*Re* = 5.0 to 252.0) (fig. S5). However, at flow rates exceeding 1250 μl/min, a gradual shift in the particle focus position is observed due to inflation of the polydimethylsiloxane (PDMS) elastomeric channels (fig. S5 and table S3).

To verify that PDMS expansion is causal to focus position deviation, rigid epoxy devices that are not susceptible to channel expansion at higher flow rates were developed (*23*, *24*). These devices have the same dimensions as the channels used in Figs. 1 and 3. As 10 μm particles flowed through the rigid device, the equilibrium focus position remained largely invariant (fig. S6 and table S4). Relative to higher flow rates (2000 and 2500 μl/min), the focusing position at 500 μl/min differs by less than 3 μm. This data, collected with a rigid epoxy device, demonstrates that the focus position is maintained at high flow rates when PDMS inflation is not a limitation. We did not test beyond 2500 μl/min due to the risk of fluid leakage. We summarize our results for the PDMS devices in a focusing regime map that shows the focusing status as a function of Reynolds number and confinement ratio (fig. S7). Finite element simulations for each tested flow rate also demonstrate the formation of flow-rate-independent transverse recirculation regions (fig. S8), and the stagnation region remains unchanged in its position. A high-speed camera video shows 10 μm particles precisely aligned in a streamline at 500 μl/min (movie S2). These computational and experimental results demonstrate that RAMP can focus particles in a flow-rate-insensitive manner in the *x*-*y* plane over a 25-fold change in flow rate, with minimal dispersion in the equilibrium position of particles.

Next, we examined the effect of varying flow rate on particle focus position in the *z*-direction (Fig. 3C). As measured from the bottom of the channel, the particle focusing positions for each flow rate tested ranged from 30.6 ± 0.6 to 32.4 ± 0.5 μm (Fig. 3D and table S5). These results demonstrate that RAMP achieves single-streamline focusing in all three dimensions (*x*, *y*, and *z*), a capability not afforded by the most inertial focusing devices, such as rectangular channels (*21*), spiral focusing (*25*), or asymmetric serpentine channels (*26*), which typically yield two focusing positions in the *z*-direction. This 3D single-line focusing feature will be particularly useful for imaging flow cytometers, as it will minimize out-of-focus events.

To ensure that RAMP’s precise flow-rate insensitive focusing capabilities extend to polydisperse particles, we tested 10 μm, 15 μm, 20 μm, and 30 μm particles at flow rates ranging from 250 to 1000 μl/min (*Re* = 25.2 to 100.8 and confinement ratios of 0.12, 0.18, 0.24, and 0.36). High-speed streak images of particles at 250 μl/min show particles focusing in approximately the same position (Fig. 3, E and F). Particles of various diameters (10 μm, 15 μm, 20 μm, and 30 μm) had average focus positions ranging from 54.2 ± 1.3 to 59.5 ± 2.3 μm (table S3). A high-speed camera video shows 10 and 30 μm particles flowing at 500 μl/min through a RAMP device (movie S3), producing identical focused streams. This consistency in polydisperse particle focusing was also observed at flow rates of 500, 750, and 1000 μl/min (fig. S9 and table S3). These analyses demonstrate that a RAMP device can tightly focus polydisperse particles across a wide flow rate regime with minimal variation in focus position.

### RAMP design parameters

**Fig. 4.**
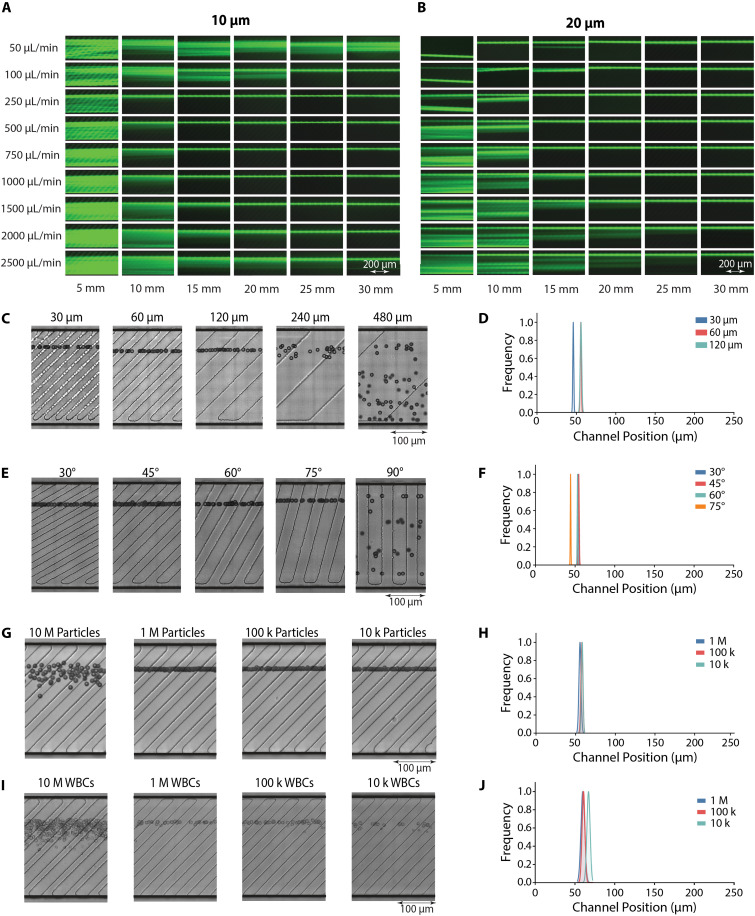
Key design parameters in RAMP devices. (**A** to **B**) Channel length required for equilibrium inertial focusing at various flow rates for 10 μm (A) and 20 μm (B) particles. (**C** to **J**) Effect of ridge wavelength, angle, and cell concentration on focusing. These experiments were performed with 10 μm particles and peripheral blood mononuclear cells (PBMCs) at a flow rate of 500 μl/min (*Re* = 50.4). (C) Effect of ridge wavelength on inertial focusing. (D) A frequency distribution diagram of particle centroid position at ridge wavelengths of 30 (*n* = 455), 60 (*n* = 367), and 120 μm (*n* = 381). (E) Effect of the ridge angle on inertial focusing. (F) A frequency distribution diagram of particle centroid position at ridge angles of 30° (*n* = 174), 45° (*n* = 402), 60° (*n* = 415), and 75° (*n* = 395). (G) High-speed streak imaging shows that 10 μm particles maintain their focus position across various concentrations, except at 10 million particles/ml, where broadening of the focus position is observed due to particle-to-particle interactions. (H) A frequency distribution diagram of particle centroid positions at particle concentrations of 1 million/ml (*n* = 18,543), 100 thousand/ml (*n* = 1937), and 10 thousand/ml (*n* = 190). (I) Similarly, high speed streak imaging of PBMCs demonstrates focus position maintenance, except a dispersion at a higher concentration of 10 million cells/ml, and (J) frequency distribution diagram quantifies focus position at cell concentrations of 1 million/ml (*n* = 391), 100 thousand/ml (*n* = 92), and 10 thousand/ml (*n* = 24).

The length of the channel required for equilibrium inertial focusing (*L_eq_*), the ridge microstructure wavelength (λ) and angle (θ), and the particle or cell concentration can all impact RAMP capabilities. We investigated equilibrium channel length for 10 and 20 μm particles using epifluorescence imaging across 50 μl/min to 2500 μl/min (*Re* = 5.0 to 252.0) (Fig. 4, A and B). A channel length of 30 mm is more than sufficient to focus particles across the range of 100 μl/min to 2000 μl/min. At a lower *Re* (∼5), inertial forces are weaker, requiring a long channel length for focusing, whereas at a higher *Re* (>25), equilibrium channel length again increases. This can be attributed to inflation of elastomeric PDMS channels, which lowers the confinement ratio, consequently reducing inertial forces and increasing the equilibrium channel length (fig. S10).

The ridge wavelength (λ) specifies the spacing between adjacent ridges (fig. S1A). Devices with ridge wavelengths of 30, 60, 120, 240, and 480 μm were created to assess the resulting particle focusing (Fig. 4, C and D, and table S6). Due to mylar mask limitations, ridges with a 30 μm wavelength could not be resolved adequately during photolithography, whereas larger ridges were produced with good fidelity. The focus position remained consistent across the 60- and 120-μm ridge wavelengths and deteriorated from 240 to 480 μm. This is attributed to a decrease in secondary flow as the wavelength increases, leading to a loss of stable focusing. The inverse relationship between secondary flow (*u’*) and wavelength (λ) is captured in eq. S4. Based on these observations, we selected a wavelength of 60 μm throughout this text.

The ridge angle (θ) is another design variable that affects the equilibrium focusing status (fig. S1A). We tested ridge angles from 30° to 90°. Ridges at angles of 15° or less were harder to resolve due to mylar mask limitations (Fig. 4, E and F). Based on eq. S4, the fluid velocity along the ridges is proportional to cos(θ) and will vanish when the ridge angle is 90°. Stable focusing was observed when ridges were angled at 30°, 45°, and 60° (Fig. 4, E and F). At a 75° ridge angle, a focus position shift of approximately 10 μm was observed, owing to the reduction in secondary flow (Fig. 4, E and F, and table S7). When ridges are angled at 90°, commensurate with theory (eq. S4), the transverse vortex that facilitates equilibrium focusing is no longer generated, and particles are not focused. Based on these observations, we used ridges at a 45° angle throughout this work.

Cell concentration capacity is another important parameter. To ascertain this, we tested particle and cell concentrations over four orders of magnitude at 500 μl/min (*Re* = 50.4): 10 thousand/ml, 100 thousand/ml, 1 million/ml, and 10 million/ml (Fig. 4, G to J, and table S8). Particle suspensions with concentrations of 10 thousand/ml to 1 million/ml were aligned within a tight 3 μm range (Fig. 4, G and H, and table S8). At a particle concentration of 10 million/ml, due to crowding, particle-particle interactions disrupted the focusing into a band around the equilibrium position. In suspensions with cell concentrations of 10 thousand/ml to 1 million/ml, alignment was observed within an 8 μm range (table S8). Higher dispersion was observed at a cell concentration of 10 million/ml, which can again be attributed to cell-cell interactions. In summary, the RAMP device can align cells over a wide range of concentrations, though focusing quality is lower when processing suspensions at 10 million/ml.

**Fig. 5.**
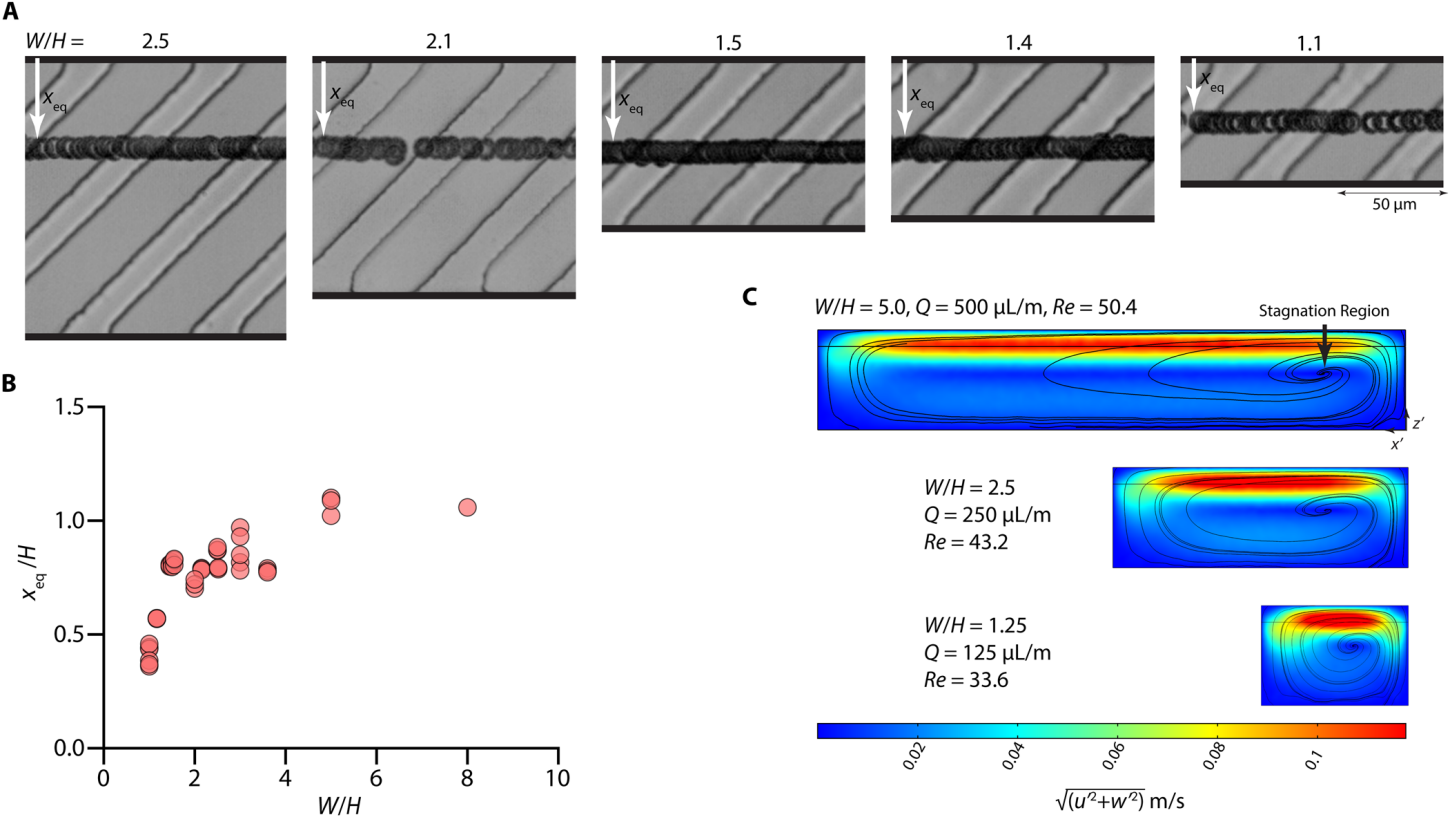
The RAMP focusing mechanism is independent of channel width for low aspect ratio channels (W/H >> 1). (**A**) The equilibrium focus position (*x_eq_*) remains constant as 10 μm particles flow through channels of varying widths (unless *W* ≈ *H*). (**B**) A scatter plot displays average equilibrium focus positions of particles flowing in channels of various aspect ratios. (**C**) Finite-element fluid flow simulations show color-mapped transverse velocity magnitudes and black streamlines for three channels of varying width and flow rate. In these simulations, the position of the stagnation region where trapping occurs remains unchanged, unless *W* ≈ *H*.

Another versatile feature of the RAMP platform is that the focus position is weakly dependent on the channel cross-sectional width for low-aspect-ratio channels (*W/H >>* 1) (Fig. 5). Particles flowing through channels with an aspect ratio of 2.5 to 1.4 focus in the same equilibrium position (labeled *x_eq_*) (Fig. 5, A and B). However, larger aspect ratio channels (*W/H* = 1.1) create equilibrium positions closer to the center of the channel (Fig. 5, A and B). Finite element simulations show the transverse velocity field in a cross-section parallel to ridge microstructures for three channels, with each successive channel half the width and half the flow rate of the one above it, starting from 250 μm and 500 μl/min (Fig. 5C). Channel and ridge heights remain constant at 50 μm and 10 μm, respectively. For W/H >> 1, the flow field and stagnation point stay the same, contributing to the equilibrium position consistency observed experimentally; however, when *W* ≈ *H*, the stagnation point shifts toward the channel center. This analysis shows that the equilibrium position remains stable in low-aspect-ratio channels ($x_{\mathrm{eq}}\approx 1.1H$) and provides insight into channel-geometry considerations.

### RAMP for cell concentration

**Fig. 6.**
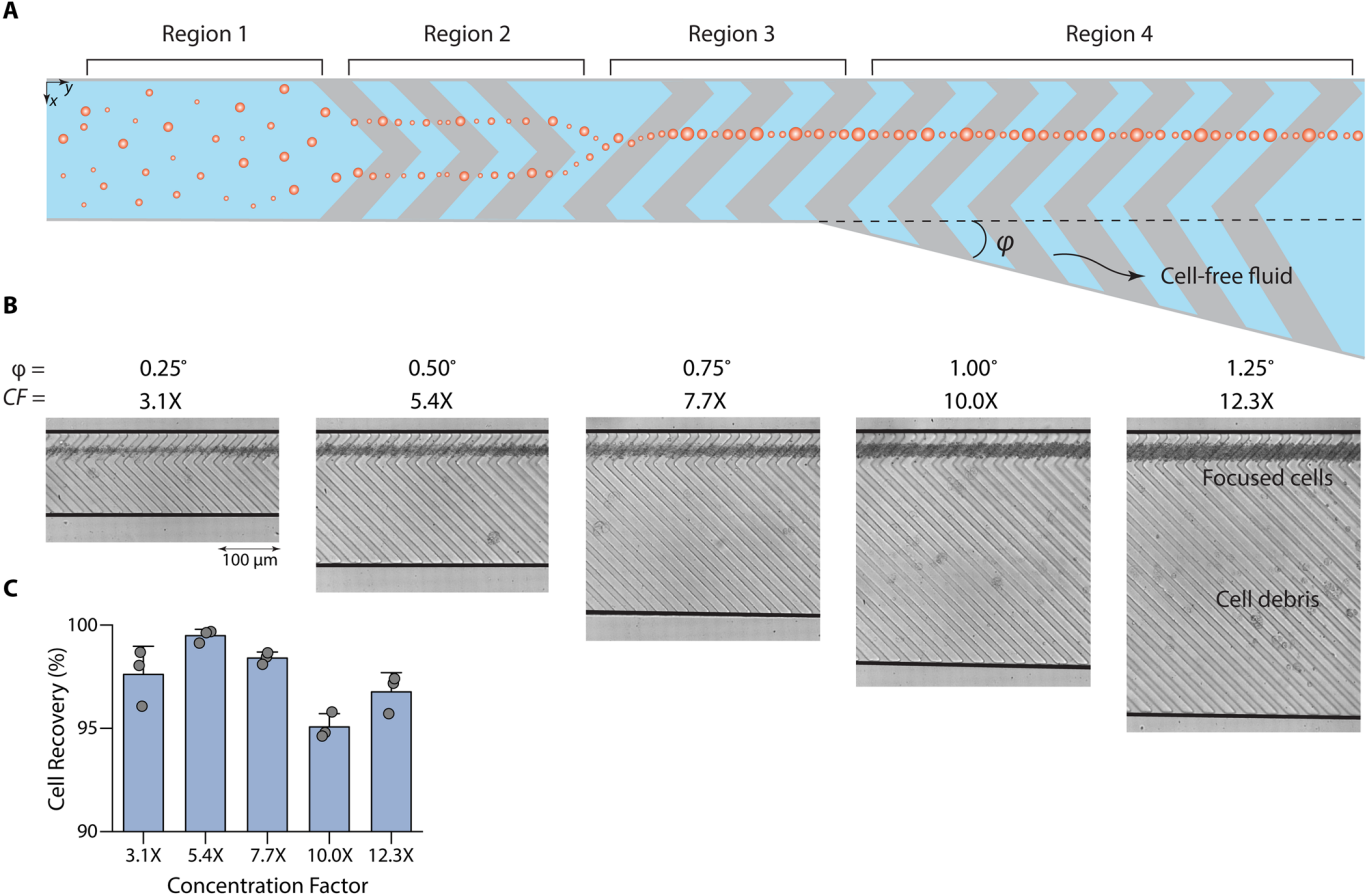
Microfluidic concentration of cells and polydisperse particles using RAMP. (**A**) A schematic representation of a concentrator with RAMP illustrates the focusing mechanism applied for concentration: region 1 shows particles entering the device, region 2 shows focusing of particles with symmetrical ridges that bring particles to the center of the channel in two streamlines, and region 3 is a long, single ridge that aligns particles in a single line near the center of the channel. Region 4 features a siphoning region that facilitates the removal of cell-free fluid, thereby concentrating cells. (**B**) RAMP concentrators enrich a mixture of PBMCs and circulating tumor cells at concentration factors of 3.1X to 12.3X. (**C**) Cell recovery at various concentration factors (*n* = 3).

RAMP’s practical benefits for sample preparation are exemplified through its use in cell concentration. Existing microfluidic concentrators often utilize free-standing posts that trap cellular debris or neutrophil extracellular traps (NETs). As a result, they are susceptible to clogging and compromised device performance (*27*). Using the RAMP concept, we developed a concentrator for reliable on-chip concentration of polydisperse particles without any free-standing posts. In this concentrator device, we exploit three key features of RAMP: (i) focusing position consistency despite changes in the average velocity of the fluid flow as the cross section increases, (ii) the alignment of polydisperse cells to identical focusing positions, and (iii) the ability to align particles into specific streamlines by changing the ridge location. A suspension of cells or particles enters the concentrator (Fig. 6A, region 1), where cells are progressively organized through three alignment regions. First, a 100 μm wide and 50 μm deep channel with chevron microstructures aligns cells into two symmetrical streamlines (Fig. 6A, region 2). Then, the two symmetrical streamlines converge into a single streamline as cells enter a third region with single ridge microstructures (Fig. 6A, region 3). Once aligned, cells retain their focus position, even as the bottom region of the channel is expanded with the siphoning angle (φ), allowing cell-free fluid to be efficiently removed in the siphoning layer (Fig. 6A, region 4). High-speed images of particles in regions 1–4 of the concentrator demonstrate the stepwise concentration principle developed using RAMP (fig. S11A). We can achieve desired concentration factors by increasing the siphoning angle.

We tested concentrators with a mixture of peripheral blood mononuclear cells (PBMCs) and circulating tumor cells (MGH-BRx-142) (Fig. 6B) and with a mixture of 10- and 20-μm particles (fig. S11B and movies S4 to S7). When mixed cell populations were processed through the concentrators, concentration factors of 3.1, 5.4, 7.7, 10.0, and 12.3 yielded average cell recoveries of 97.6 ± 1.1%, 99.5 ± 0.2%, 98.4 ± 0.2%, 95.1 ± 0.5%, and 96.8 ± 0.8%, respectively (Fig. 6C and table S9). For polydisperse particle suspensions, recoveries remained consistently high at 99.7 ± 0.2%, 99.8 ± 0.1%, 99.7 ± 0.2%, 99.0 ± 0.2%, 99.6 ± 0.1%, and 99.9 ± 0.1% across concentration factors of 3.1, 5.4, 7.7, 10.0, 12.3, and 20.8 (fig. S11C and table S9). Overall, particles and cells exhibited excellent yields, resulting in more than 95% cell recovery with concentration factors ranging from 3.1 to 20.8, including polydisperse suspensions and cell populations. Minor cell losses in the mixed cell population can be attributed to dead cells or debris in the suspension, labeled in Fig. 6B. When we increased the siphoning angle to 5°, the particle stream diverted into the siphoning region, so RAMP concentrators should be designed with φ < 5° (fig. S11B).

### Additional RAMP applications

**Fig. 7.**
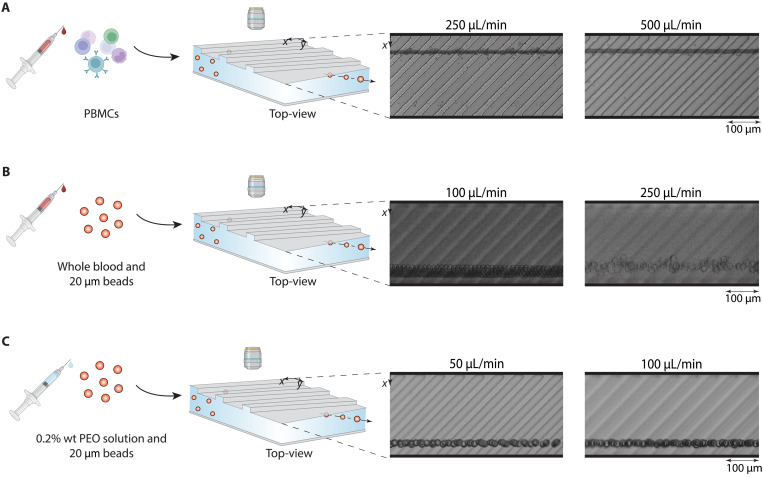
RAMP facilitates robust focusing across diverse sample types, including whole blood and viscoelastic fluids. (**A**) RAMP focuses peripheral blood mononuclear cells (PBMCs) dispersed in a buffer. (**B**) RAMP can focus particles in undiluted whole blood. Here, we used 20 μm particles to more clearly visualize the focusing effect, as they are readily visible during high-speed imaging (movie S8). (**C**) RAMP achieves viscoelastic focusing of particles in a polyethylene oxide (PEO) solution. Created in BioRender. Payan-medina, A. (2026) https://BioRender.com/i4yx21j.

RAMP enables focusing across a range of sample types, including PBMCs in buffer (Fig. 7A), whole blood (Fig. 7B), and viscoelastic polymer solutions (Fig. 7C). Various liquid biopsy tests often use whole blood and PBMCs as the primary fluid (*28*–*30*). We demonstrate that the RAMP device can align PBMCs at flow rates ranging from 250 to 1000 μl/min (Fig. 7A and fig. S12A), thereby enabling the interrogation of clinically relevant human samples. Further, we show sustained, clog-free processing of PBMCs (fig. S13). PBMCs were flowed through a device with RAMP at 250 μl/min for 4 hours at 0.5 million cells/ml, with minimal deviation in focus position (table S10), demonstrating focus position stability without channel clogging, cellular adhesion, or deposition that may inhibit processing capability.

Another essential factor to consider in sample preparation is the ability to focus cells or particles in undiluted whole blood. Due to the excessive number of red blood cells (RBCs) in the blood (5 billion RBCs/ml), hydrodynamic particle-particle interactions disrupt equilibrium positions within the channel (*17*). We added 15, 20, and 30 μm particles to whole blood and conducted experiments at flow rates of 100 and 250 μl/min. These bright, large particles enabled us to directly visualize particle focusing in whole blood despite the high RBC concentration. These particles in whole blood were still aligned near the channel sidewall, though at the opposite wall (near *x = W*) (Fig. 7B, fig. S12B, and movie S8) than the previously observed results with Newtonian fluids (Fig. 3E). This flip in the focusing position could be due to the shear-thinning viscoelastic properties of whole blood. The ability to directly focus particles or cells in undiluted whole blood is challenging for other inertial focusing methods, showcasing a remarkable property of the RAMP system that may enable higher cellular throughput and direct sorting from whole blood. Previously reported methods diluted blood 10- to 25-fold before using inertial focusing (*31*, *32*). In this case, RBCs and platelets were not focused due to their low confinement ratio (≤0.07). We could not test particles smaller than 15 μm because they are difficult to visualize in whole blood.

To test the role of viscoelastic forces in shifting the focusing position, we spiked the same 20 μm diameter particles in a 2000 ppm aqueous solution of polyethylene oxide (PEO), which imparts shear-thinning viscoelastic properties to the fluid (*33*, *34*), and tested the solutions at 50 and 100 μl/min through the RAMP channel. Viscoelastic fluids exhibit properties of both viscous liquids and elastic solids, which cause them to flow while storing energy when deformed, thereby generating elastic stresses. The combination of inertia and elasticity in a viscoelastic fluid induces microparticle focusing by introducing an elastic lift force on particles (*33*, *35*) (eq. S6 and fig. S14A), arising from the imbalanced first normal stress difference across the channel, and is highly dependent on particle size. In a rectangular channel, away from the top and bottom walls, elastic forces act towards the channel’s centerline for a shear-thinning fluid (fig. S14B). The elasticity effect is greater at higher PEO concentrations, flow rates, and particle confinement ratios (*33*).

Deborah’s number (*De*) is defined as the ratio of fluid relaxation time to characteristic time of the deformation process (*36*, *37*) (eq. S7). A medium will exhibit fluid-like behavior when the characteristic time is large or the relaxation time is small, and solid-like behavior if the characteristic time is small or the relaxation time is large (*36*). Inertial effects can be quantified using the Reynolds number (eq. S8). Elastic and inertial effects can be compared using the Elasticity number (eq. S9), which is the ratio of elastic to inertial forces. The Deborah number, Reynolds number, and the Elasticity number for each flow condition tested are detailed in table S11. Since the Elasticity number is >100 at both flow rates, elastic forces dominate over inertial forces, confirming viscoelastic focusing as the primary focusing mechanism. In this regime, particles are concentrated toward the centerline of the rectangular channel (fig. S14B), where the transverse flow field is directed towards the far end of the ridge (fig. S14C), causing particles to be swept and focused at a position closer to the distant sidewall (near *x = W*). We hypothesize that, at this position, elastic force (*F_E_*) balances the drag force (*F_D_*) (fig. S14D and eq. S10). We limited testing to 100 μl/min as PDMS channels start to inflate at higher flow rates due to increased pressure drop caused by highly viscous viscoelastic fluids. Here, it is evident that particles retain the precise focusing effect of RAMP in viscoelastic fluids, and just as in whole blood, the particle focus position is observed at the far end of the ridge, suggesting that the focusing in undiluted whole blood may be controlled by viscoelastic forces (Fig. 7B). Thus, RAMP can achieve stable, deterministic focusing in undiluted whole blood and in viscoelastic fluids.

## DISCUSSION

Handling complex cellular biofluids involves aligning target cells into specific locations for isolation, characterization, or analysis to improve the sensitivity and efficiency of detection methods (*38*). For example, high-gradient magnetic sorting demands that cells be positioned near channel sidewalls, where magnetic gradients are strongest (*39*), and plasmonic or whispering-gallery mode lasers or sensors require cells to pass close to sensing features for effective signal generation (*40*). Similarly, imaging flow cytometry requires cells to be focused tightly in a single streamline in all three dimensions (*41*). Sample preparation of cells in biofluids is complicated by the cell size variability, which may vary across patients or within a single patient. Several biofluids, such as peritoneal and pleural fluids, also contain cellular debris and byproducts that can form channel blockages around pillars in the channel. In this work, using rationally designed ridge arrangements, we have described a RAMP method that can focus particles and cells to any desired lateral position in a channel, applicable to a wide range of particle sizes, flow rates, and channel widths. We have leveraged these unique features to engineer a clog-free cell concentrator and to focus particles in undiluted whole blood.

Several microfluidic devices have been developed to accommodate and prepare diverse cell populations for downstream analyses (*13*, *30*, *39*). Active microfluidic techniques use external forces and fields, such as electric or magnetic fields, to focus particles (*42*, *43*). Passive microfluidic methods, including inertial focusing, can order particles more simply using channel microstructures. Microfluidic inertial focusing facilitates high-throughput particle control in a fluid stream by utilizing hydrodynamic inertial lift forces and secondary flows (*17*). Several microchannel configurations have been designed for inertial focusing, including serpentine, reverse-wavy, contraction-expansion, spiral, and slanted groove designs (*42*, *44*–*47*) (table S1). These inertial focusing devices have been especially successful for separating cells based on size (*48*, *49*) (table S1). In the past, ridge microstructures have also been used to manipulate particles and cells (*50*–*58*). Physical interaction with antibody-coated ridges and channels has been used to capture cells or to alter their trajectory through transient molecular interactions (*53*–*55*). Physical compression of cells in channels with periodic ridges has been elegantly used to sort cells based on their stiffness and to porate them for gene delivery (*56*–*58*). However, all of these approaches align cells along different streamlines across the channel cross-section, creating size- or deformability-based dispersion. Overall, the particle equilibrium position cannot be controlled with any of these methods.

RAMP enables three-dimensional particle focusing within a single stream without relying on co-flow. Though microfluidic systems that utilize sheath flow have been used (*13*, *59*, *60*) for confining cellular streams with buffer co-flow, sheath streams induce substantial shear stress, reduce throughput, and add complexity to the overall design (*61*). Some ridge-based inertial focusing techniques can focus particles across a range of flow rates (*12*, *62*). However, these systems have not demonstrated the ability to control the particle-focusing position in a channel (*15*). Remarkably, these systems also use high non-dimensional ridge heights (α=h/2H′) ranging from 25% to 47% (*12*, *50*, *63*), which differ from RAMP’s low non-dimensional ridge height of ∼5%.

In contrast, RAMP is an adaptable, sheathless, high-throughput technique that allows tunable alignment of polydisperse cells and particles. We demonstrate precise tuning of the focus position, enabling both small (5 μm) and large (≥15 μm) shifts by adjusting the ridge microstructure within the channel. RAMP’s precise focusing capabilities extend to a wide range of particle sizes and a 25-fold change in flow rate. We observed focusing of 10–30 μm diameter particles within 2% of the width of the channel (5 μm) and 10 μm particles within 4% of the height of the channel (2 μm) in the *z*-direction. Unlike various inertial techniques that focus particles in two equilibrium positions in the *z*-direction (*25*, *26*), RAMP precisely focuses particles to one *z*-position. Manipulating cells’ 3D focus position is especially advantageous for imaging-based cytometry to minimize event dropouts, where typically only 50% of cells are focused (*64*).

We explored the effect of RAMP design parameters, such as channel length, ridge wavelength, ridge angle, and cell concentration, on the focusing performance. A 30 mm channel length was sufficient to focus 10 and 20 μm particles across most tested flow rates. However, the focusing length increased at very low Reynolds numbers and again at higher Reynolds numbers, potentially due to PDMS channel inflation. Among ridge designs, the focusing position did not change at shorter wavelengths (60–120 μm) but deteriorated at longer wavelengths, consistent with weaker secondary flows at higher ridge wavelengths. Similarly, stable focusing was maintained at ridge angles of 30° to 60°, shifted at 75°, and, consistent with theoretical work, focusing was lost at 90° due to a lack of secondary flow. RAMP also tolerated cell concentrations over four orders of magnitude, but focusing quality declined at 10 million/ml due to cell crowding at the focusing position.

As RAMP’s focusing behavior is largely unaffected by channel width, it is highly compatible with integration across multiple platforms, offering flexibility for scale-up. Further, RAMP can effectively focus PBMCs and particles in undiluted whole blood and viscoelastic fluids, demonstrating its adaptability to a wide range of biological fluids. Leveraging RAMP’s ability to focus polydisperse particles across a range of flow rates and channel widths, we developed a clog-free cell concentrator. Volume reduction is essential in biofluid processing, particularly for large volume samples such as 100 ml leukapheresis products or 1000 ml ascites samples. These samples often contain cell debris, clots, and NETs that clog devices and wrap around pillars used in previously demonstrated concentrator designs (*27*). In contrast, RAMP concentrators utilize ridge microstructures with a low non-dimensional ridge height (α≅0.05) and a predominantly featureless channel design, effectively eliminating clogging. Using this approach, we achieved concentration of polydisperse cells and clusters with >95% recovery across multiple concentration factors.

Despite these promising results, several aspects warrant further investigation. The physical basis of the RAMP focusing mechanism remains to be fully defined, particularly for cells and particles suspended in whole blood. Since RAMP focusing includes an interplay between inertial lift forces, viscoelastic forces, and ridge-induced secondary flows, future studies could simulate particle trajectories under these coupled effects. In this study, we also could not explore flow rates above 2500 μl/min due to concerns about fluid leakage. In the future, it would be interesting to see if focusing remains unchanged at higher flow rates in rigid devices. Finally, depending on the channel geometry, RAMP devices are expected to have an upper limit on the cell concentration that they can handle, as focusing performance deteriorates due to cell-to-cell interactions.

In summary, RAMP uniquely enables precise control of the lateral focusing position of cells in a microchannel across a wide range of flow rates, cell sizes, and concentrations. This capability allows effective focusing in whole blood and facilitates clog-free cellular concentrators. The development of RAMP provides an easy-to-integrate and scalable focusing method that will advance cell sensing, imaging, and sorting applications across a wide range of biofluids.

## MATERIALS AND METHODS

### Experimental design

This paper presents a microfluidic approach for tunable and precise inertial focusing across a wide range of particle sizes and flow rates. The geometric properties of the ridge microstructure were optimized to focus particles of varying sizes (10 to 30 μm) at flow rates that enable high-throughput processing (10 to 2500 μl/min). Unless specified, particles and cells were suspended at a concentration of 200,000/ml. Channel dimensions and fluid conditions were kept constant when different flow rates or particle sizes were tested. Flow and particle size conditions tested were repeated (*n* ≥ 3) to ensure reproducibility. Pressure drop through a RAMP channel for various flow rates is shown in fig. S15. The primary outcome was particle focusing position and cell recovery at various concentration factors; secondary outcomes included the standard deviation of the focusing position.

### Microfabrication

Standard lithography techniques were used to fabricate polydimethylsiloxane (PDMS) devices at Massachusetts General Hospital. A mold for PDMS devices was produced by applying SU-8 50 at 2650 rpm and SU-8 5 at 3000 rpm for 30 seconds to a prebaked silicon wafer. The objective target thicknesses for the channel layer and microstructures were 50 μm and 10 μm, respectively. Channels were produced by irradiating the SU-8 layer with 365 nm UV light through a Mylar mask. Ridge microstructures were developed by treating the exposed SU-8 layer with Baker BTS-220 SU-8 developer. PDMS devices were made using the Sylgard PDMS kit, mixed at a 10:1 base-to-crosslinker ratio, poured into the SU-8 mold, degassed in a vacuum chamber, and cured in a convection oven at 65°C for 12 hours. A 1.2 mm biopsy punch was used to drill inlets and outlets, and PDMS devices were plasma-bonded to 3-inch by 1-inch glass slides and baked at 150°C for 3 hours.

Rigid devices shown in fig. S6 were fabricated using an EpoxAcastTM 690 transparent epoxy kit (Smooth-On). Unbonded PDMS devices made from the original SU-8 mold were passivated with Trichloro(1H,1H,2H,2H-perfluorooctyl)silane and subsequently casted with a standard PDMS mixture to create flexible molds. A 2 mm biopsy punch was used to drill inlets and outlets directly into the PDMS molds. 6 mm polytetrafluoroethylene (PTFE) rods attached to 2 cm polyethylene (PE) tubing were inserted into the drilled holes before epoxy casting. The epoxy mixture was prepared by thoroughly mixing Parts A and B of the kit at a 10:3 ratio, degassing for 3 minutes, and then pouring into the PDMS molds. The cured epoxy devices were peeled from the molds 22 to 24 hours after pouring, and PTFE rods were pulled from the inlets and outlets. Epoxy devices were then bonded to plasma-treated 3-inch-by-1-inch glass slides, which were resting on a 70°C hot plate. The devices were left on the hot plate for 30 seconds and then stored at ambient temperature for 24 hours before use.

### Side-view device fabrication

To observe particle focusing in the *y*-*z* plane, a channel with ridge microstructures was bonded to a PDMS base layer. A viewing window was created by cutting close to the microchannel and base layer sidewall, leaving a thin layer of PDMS enclosing the channel. To restore transparency in the viewing window, a thin layer of uncured PDMS was poured onto a glass slide, and the cut channel was adhered to it. The sample was then baked at 80°C for 30 minutes. This fabrication process is illustrated in fig. S16.

### Particle and cell inertial focusing in microfluidic flow

A Harvard Apparatus syringe pump and Tygon thermoplastic tubing were used to flow Fluoro-Max 10 μm, 15 μm, 20 μm, and Duke Standards 30 μm fluorescent particles at flow rates ranging from 250 to 2500 μl/min through devices. A Nikon Ti-U inverted microscope, Phantom 4.2 camera (Vision Research Inc.), and QImaging Retiga 2000R CCD camera were used to capture high-speed and fluorescent images of particles and cells flowing in microchannels. COMSOL Multiphysics was used to simulate velocity fields. A 2000 ppm PEO solution was combined with Fluoro-Max particles of 15, 20, and 30 μm diameter to test RAMP’s viscoelastic focusing capabilities. Based on prior work, the dynamic viscosity and relaxation time of the 2000 ppm PEO solution were 5 × 10^−3^ Pa·s and 0.0195 s, respectively (*65*, *66*).

### CTC culture

The protocol used for MGH-BRx-142 CTC culture is reported in (*67*). MGH-BRx-142 CTCs were cultured in media with RPMI-1640, 1X B27 (Gibco), EGF (20 ng/ml) (Gibco), bFGF (20 ng/ml) (Gibco), and 1X antibiotic/antimycotic (Life Technologies) in ultralow attachment flasks (Corning). Then, several rounds of manual pipetting were done to dissociate cells. CTC culture was performed at 37°C, under hypoxic conditions (4% O_2_), and with 5% CO_2_.

### PBMC collection

Blood was obtained from Research Blood Components, LLC, or healthy internal donors for experiments involving whole blood. Experimental protocols were reviewed and authorized by the Massachusetts General Hospital to obtain informed consent for internal whole-blood donations under Protocol 2009-P-000295. Whole blood was processed through the CTC-iChip to separate PBMCs from red blood cells and platelets into a 0.2% F68 PBS Buffer.

### Image analysis

Particle tracking was performed using ImageJ software. The workflow follows this sequence: (i) cropping and adjusting the stack of frames to only include the channel, (ii) reduction of the stack (by a factor of ∼3) to ensure particles are counted once, (iii) inversion of the stack, (iv) subtraction of an average intensity frame of the stack from each frame of the main sequence, (v) adjustment of brightness and contrast to enhance particle visibility while minimizing noise, (vi) conversion of the video to a 8-bit and binary form, (vii) adjustment of the threshold for optimal contrast between particles and background, (viii) application of erosion and dilation to the particles to refine outlines and eliminate residual noise, and (ix) determination of particle centroid positions using ImageJ’s *Analyze Particles* feature. Following ImageJ analysis, the resulting file containing the particle area and center coordinates was processed for statistical analysis.

### Statistical analysis

Particle position data were analyzed with Python Pandas and Numpy packages. The mean focusing position and its standard deviation were calculated.

## Supplementary Materials

### The PDF file includes:

Supplementary Text Figs. S1 to S16 Tables S1 to S11 Legends for movies S1 to S8 References
