# Supplementary material for: Ridge-assisted micro positioning of cells in a channel
Source: Sci Adv. 2026 Jul 17;12(29):eaec9227. doi: 10.1126/sciadv.aec9227 (PMC13378550; doi:10.1126/sciadv.aec9227)
Supplement: Supplementary file 1 — Supplementary Text Figs. S1 to S16 Tables S1 to S11 Legends for movies S1 to S8 References [file sciadv.aec9227_sm.pdf]

Science Advances
AAAS

Supplementary Materials for

Ridge-assisted micro positioning of cells in a channel

Adriana Payan-Medina *et al.*

Corresponding author: Avanish Mishra, amishra2@mgh.harvard.edu

Sci. Adv. **12**, eaec9227 (2026)
DOI: 10.1126/sciadv.aec9227

The PDF file includes:

Supplementary Text
Figs. S1 to S16
Tables S1 to S11
Legends for movies S1 to S8
References

Other Supplementary Material for this manuscript includes the following:

Movies S1 to S8

Supplementary Text

Theoretical model of fluid flow in a RAMP channel

Focusing in a RAMP device is governed by a balance of secondary-flow-induced drag force and inertial lift force. Since trapping is occurring away from the top and sidewalls, we can approximate the lift force as

Equation S1.

$$F_L = C_L \rho U^2 a^4 / D_h^2$$

where C_L is the lift coefficient, ρ is the fluid density, U is the average velocity, a is the particle diameter, and D_h is the hydraulic diameter of the channel. At the focusing positions, the inertial lift force is balanced by the secondary-flow-induced drag force in the *z*-direction, which can be written as

Equation S2.

$$F_D = 3\pi\mu a w$$

where *w* is the *z*-component of velocity and μ is the dynamic viscosity of the fluid. The lift-to-drag force ratio can be written as **Equation S3**.

Equation S3.

$$\frac{F_L}{F_D} = \frac{C_L \rho U^2 a}{3\pi\mu w}\left(\frac{a}{D_h}\right)^2$$

Theoretical treatment of secondary flow induced by ridges has been presented in previous studies (*20*). Following their analysis for flow in thin channels ($W >> H$) and shallow grooves ($h/H << 1$), the fluid velocity along the ridges (**Figure S1**) can be written as

Equation S4.

$$u' = 6\alpha^2 U\left(\frac{3}{2}\frac{(H'-z)(z)}{H'^2} - \frac{z}{2H'}\right)\left(\frac{2\pi H'}{\lambda} - 1\right)\mathrm{Cos}\,\theta$$

In **Equation S4,** λ is the wavelength, θ is the angle of the ridges, $H' = H + h/2$, and α is a geometrical nondimensional number. A relation for ridge height (h) is presented as $h = 2\alpha H'$. **Equation S4** describes the key parameters that affect secondary flow in RAMP devices. As expected, ridge-induced secondary flow scales quadratically with α and linearly with *U*. The secondary flow vanishes when ridges are placed at 90º to the main channel flow. Similarly, following the treatment in (*20*), the approximate streamwise velocity, *v*, can be written as

Equation S5.

$$v = 6U(1 - \frac{3}{2}\alpha^2(1-\widetilde{K}))\left(\frac{z\left(H' - z - \tilde{z}_{eff}\right)}{H'^2}\right)$$

where

$$\widetilde{K} = \left(-1 + 2\sigma\frac{\sinh(\sigma)\cosh(\sigma) - \sigma}{\sinh^2(\sigma) - \sigma^2}\right)\sin^2\theta + \left(-1 + \sigma\frac{\cosh(\sigma)}{\sinh(\sigma)}\right)\cos^2\theta$$

$$\tilde{z}_{eff} = \frac{1}{2}H'\alpha^2\left[\left(-1 + 2\sigma\frac{\sinh(\sigma)\cosh(\sigma) - \sigma}{\sinh^2(\sigma) - \sigma^2}\right)\sin^2\theta + \left(-1 + \sigma\frac{\cosh(\sigma)}{\sinh(\sigma)}\right)\cos^2\theta\right]$$

Here, $\sigma = 2\pi H'/\lambda$. **Equation S5** will be used to validate our computational model in the next section.

Some of the key equations for viscoelastic fluids are described below.

Elastic lift force: Elastic lift force is proportional to normal stress (N_1) variation over the particle volume (a^3). C_e represents the elastic lift coefficient.

Equation S6.

$$F_e = C_e a^3 \nabla N_1$$

Deborah's number (De): De is a ratio of the characteristic time of the fluid (τ) and the characteristic time of the deformation process (t_f).

Equation S7.

$$De = \frac{\tau}{t_f} = \frac{\tau Q}{H^3}$$

Reynolds number (Re): Re is used to compare fluid inertial and viscous forces. Fluid density, average velocity, channel hydraulic diameter, and viscosity are ρ, U, D_h, and μ, respectively.

Equation S8.

$$Re = \frac{\rho U D_h}{\mu}$$

Elasticity number (El): El is a ratio of Deborah's number divided by Reynolds number and can be used to compare a fluid's elastic and inertial properties.

Equation S9.

$$El = \frac{De}{Re}$$

The ratio of elastic lift force and drag force can be written as **Equation S10**, denoting a weaker dependence on particle size ($\propto a^2$) than the ratio of inertial lift force and drag force ($\propto a^3$).

Equation S10.

$$\frac{F_e}{F_D} = \frac{C_e a^3 \nabla N_1}{3\pi\mu a w} = \frac{C_e a^2 \nabla N_1}{3\pi\mu w}$$

Computational modeling of fluid flow in a RAMP channel

Steady-state incompressible fluid flow simulations were conducted in COMSOL Multiphysics using the Navier–Stokes equations to model ridge-induced secondary flow. A schematic diagram of the boundary conditions is shown in **Figure S1A**. A velocity inlet boundary condition was applied at the inlet, an atmospheric pressure boundary condition was applied at the outlet, and a no-slip boundary condition was imposed on the rest of the surfaces. A free tetrahedral mesh was used to discretize the model. The minimum and maximum element sizes of the mesh were varied from 0.5 to 2 µm and 5 to 20 µm, respectively, to ensure the results were independent of mesh element size, as shown in **Figure S1B**. We used a minimum mesh size of 0.5 µm (Finer Mesh) as it was computationally tractable. We used an algebraic multigrid solver based on the generalized minimal residual method.

As discussed in the theory section, approximate theoretical solutions to secondary flow (**Equation S4-S5**) produced by ridges in a rectangular channel have been presented in the previous work (*20*). To validate the computational model, we compared the theoretical streamwise velocity, v, in a RAMP channel (**Figure S1A**) at a flow rate of 500 µL/min with computational results. The theoretical velocity profile, estimated using **Equation S5***,* is in close agreement with the computational result (**Figure S1C**). The theoretical peak streamwise velocity is estimated to be 0.93 m/s, whereas the simulation result is 1.11 m/s (**Figure S1C**). It is important to note that the theoretical solution (**Equation S5)** is an approximate result for thin channels and shallow grooves, whereas the numerical solution is a more accurate result. Nonetheless, this concordance between the two results provides a validation of our computational model.

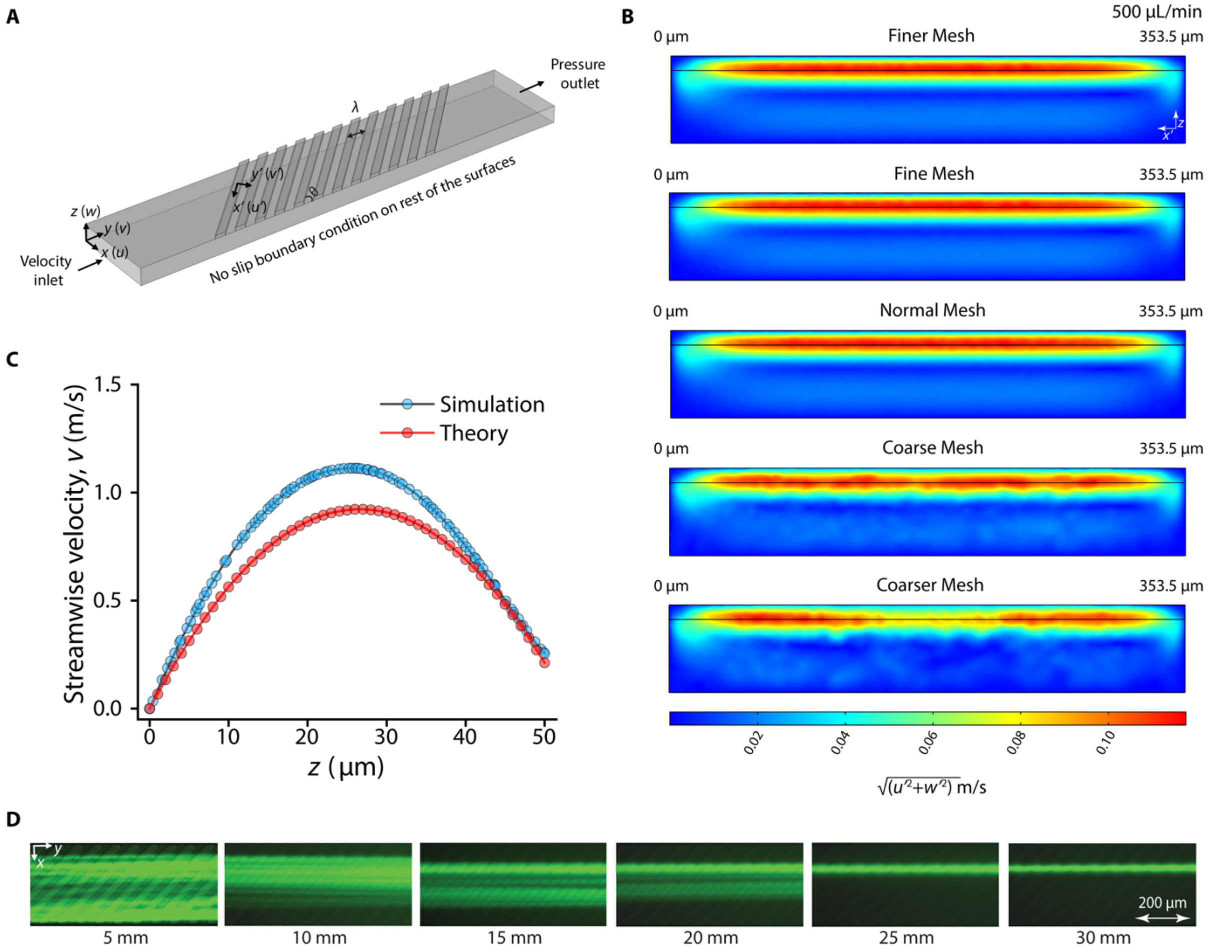

Fig. S1. Computational modelling of fluid flow in a ridged channel. (**A**) Boundary conditions used in the computational model. (**B**) Mesh refinement study. The contour plot of the secondary flow produced by ridges becomes independent of the mesh as the minimum element size is reduced to 0.5 µm (finer mesh) and 1 µm (fine mesh). (**C**) Comparison of streamwise velocity, *v*, estimated using an approximate theoretical model (**Equation S5**) and computational model at 500 µL/min (*Re* = 50.4). (**D**) Gradual focusing of 10 µm green-fluorescent particles at 100 µL/min (*Re* = 10.1) as a ridge-induced microvortex sweeps them across position-1 at the far end of the channel to the stable focusing position-2.

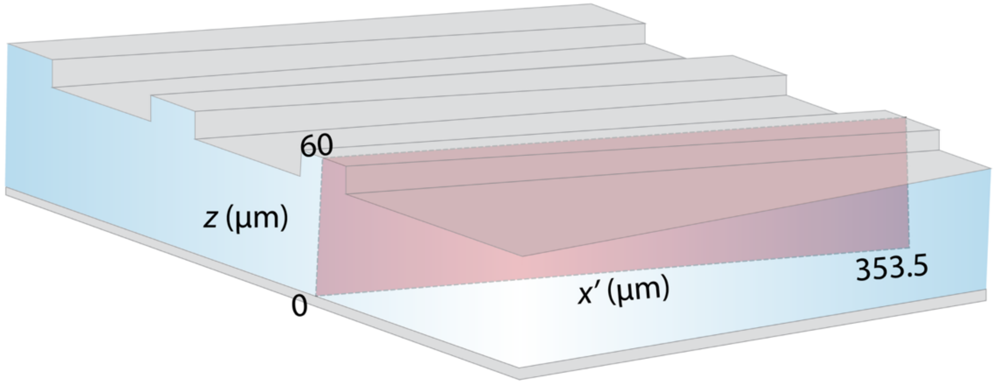

Fig. S2. A schematic representation of a RAMP device. It shows the cross-section parallel to the ridge microstructures used to present the velocity field and the microvortex.

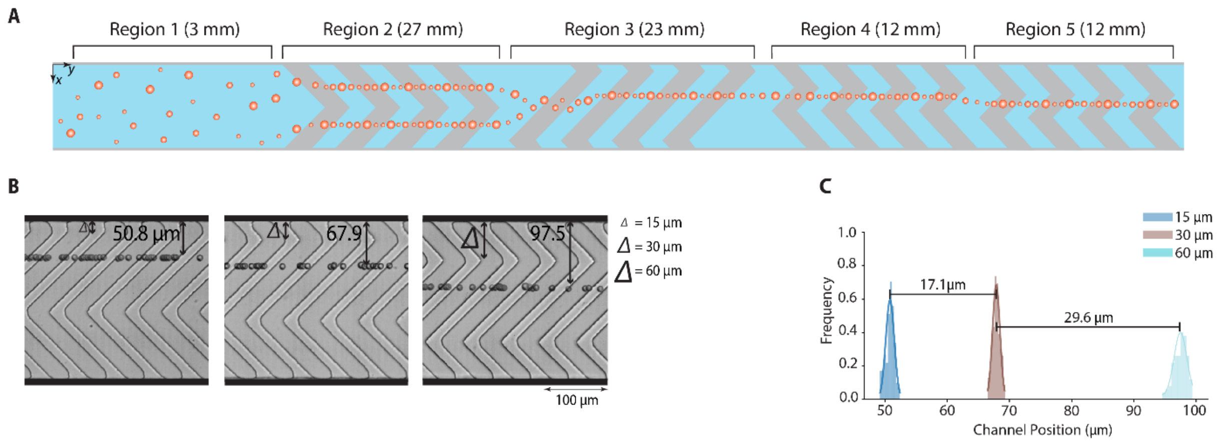

Fig. S3. Large focusing position shifts in 10 µm particle streamlines are achieved with proportional shifts in the position of the central ridge. (**A**) Length of different regions. (**B**) Shifts in particle focus position are observed as the central ridge structure is shifted in steps of 15 µm and 30 µm (image for *Δ*=60 µm is also presented in Figure 2). (**C**) A frequency distribution plot of particle centroid positions shows the focus position shift as the central ridge position (*Δ*) is shifted (15 µm (n = 131), 30 µm (n = 83), and 60 µm (n = 105)).

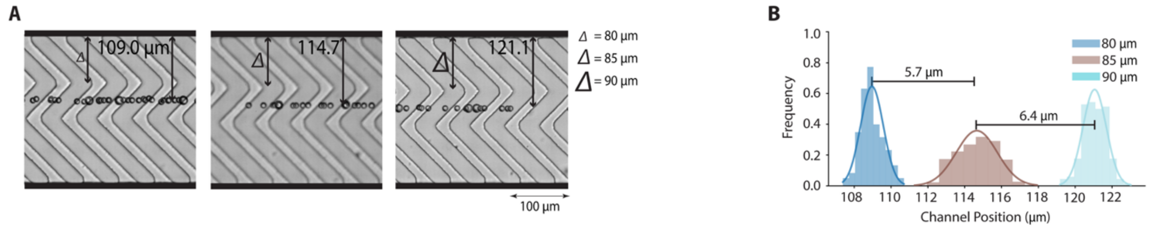

Fig. S4. Focus position shifts for a mixture of 10 µm and 15 µm particles. (**A**) Shifts in particle focus position are observed using high-speed streak imaging as the position of the central ridge structure is shifted in 5-µm increments from the channel sidewall. (**B**) A frequency distribution plot of particle centroid positions shows the focus position shift as the central ridge position (*Δ*) is shifted 80 µm (n = 384), 85 µm (n = 403), and 90 µm (n = 191) from the channel sidewall.

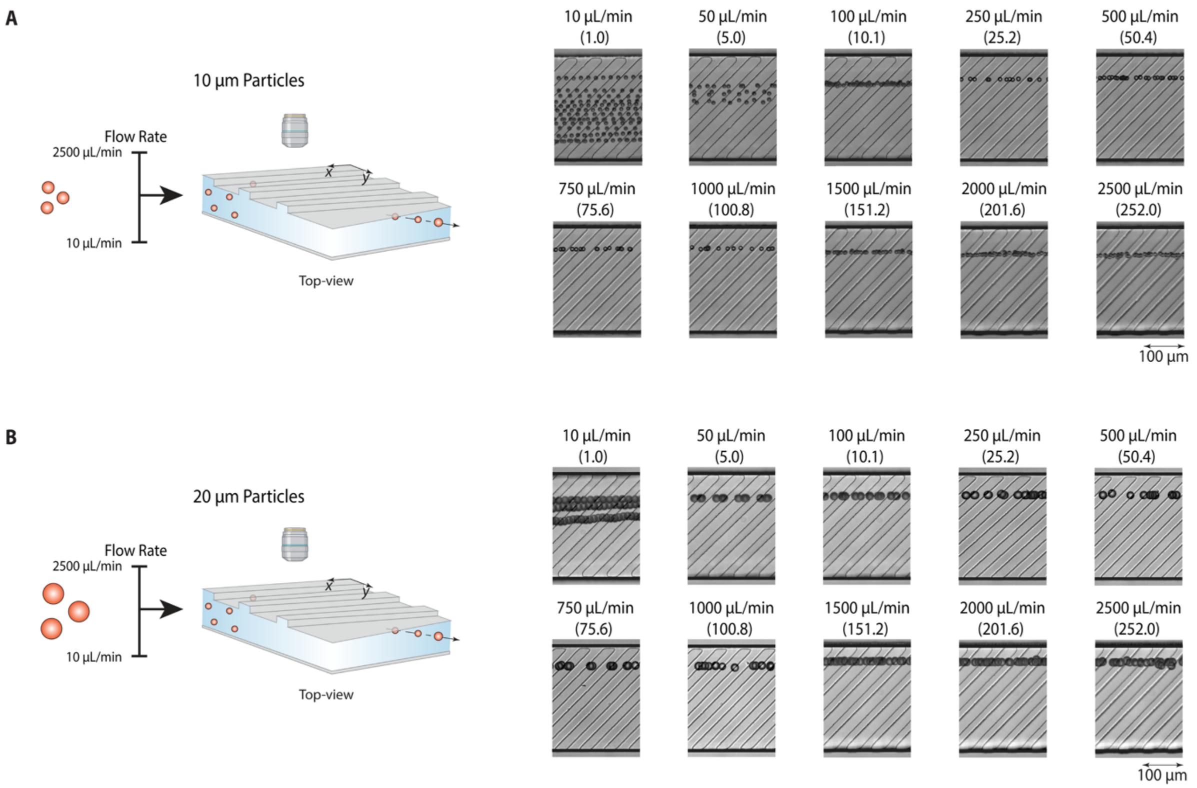

Fig. S5. RAMP focusing of particles across a wide range of flow rates. (**A**) High-speed streak imaging of 10 µm particles across a flow rate range of 10 to 2500 µL/min. Particles focus over a range of 100 to 2500 µL/min (*Re* = 10.1 to 252.0); however, at higher flow rates (1500 to 2500 µL/min), a shift in the particle focus position is observed due to inflation of the polydimethylsiloxane (PDMS) channels. Images for flow rates of 250 to 1000 µL/min were repurposed from Figure 3 for comparison across a wide flow rate range. (**B**) High-speed streak imaging of 20 µm particles across a flow rate range of 10 to 2500 µL/min. Particles focus over a range of 50 to 2500 µL/min (*Re* = 5.0 to 252.0). At higher flow rates (1500 to 2500 µL/min), a shift in the particle focus position is observed due to inflation of the PDMS channels. Channel Reynolds number is shown in parentheses below the flow rate. Flow rates above 2500 µL/min were not tested due to the risk of fluid leakage. Created in BioRender. Payan-medina, A. (2026) https://BioRender.com/i4yx21j.

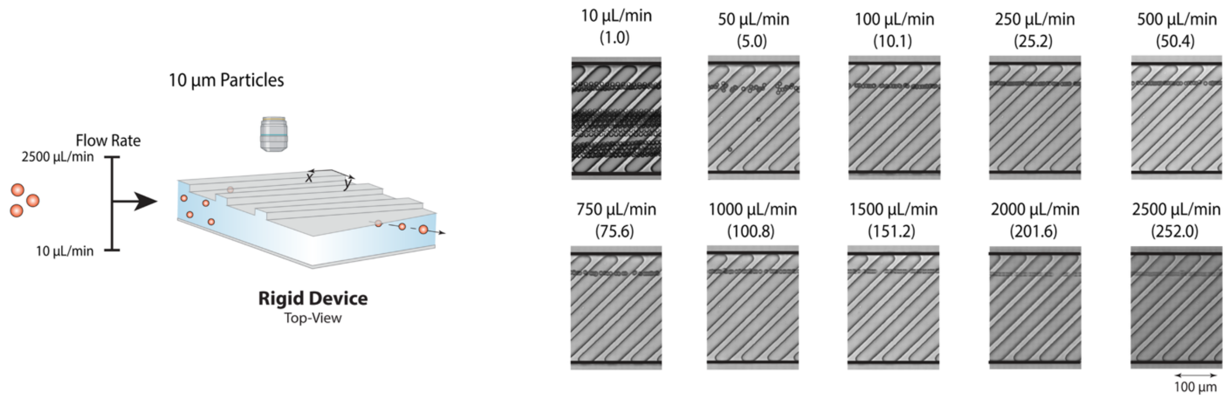

Fig. S6. RAMP focusing of particles in a rigid device. High-speed streak imaging of 10 µm particles in a rigid epoxy device over a flow rate range of 10 to 2500 µL/min. Particles focus over a range of 50 to 2500 µL/min (*Re* = 5.0 to 252.0). In these rigid devices, the focusing position remains unchanged even as the flow rate exceeds 1500 µL/min, as channels don't inflate. Flow rates above 2500 µL/min were not tested due to the risk of fluid leakage. The channel Reynolds number is shown in parentheses below the flow rate. Created in BioRender. Payan-medina, A. (2026) https://BioRender.com/i4yx21j.

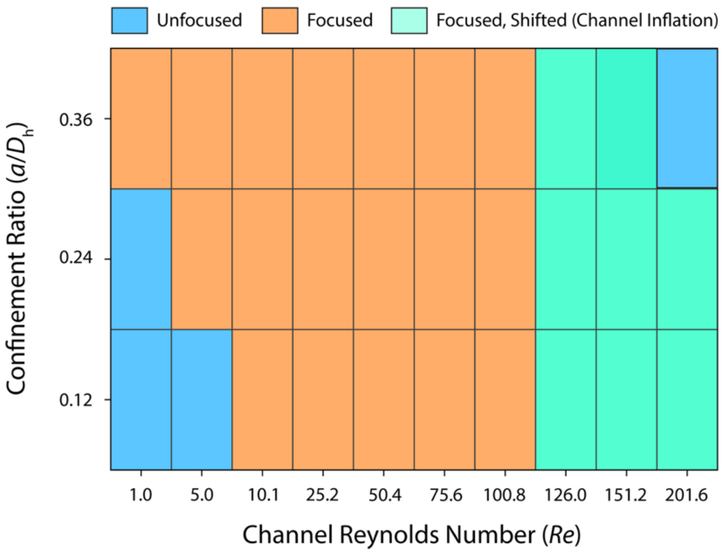

Fig. S7. Focusing regime map. Focusing status as a function of Reynolds Number and the confinement ratio for PDMS channels.

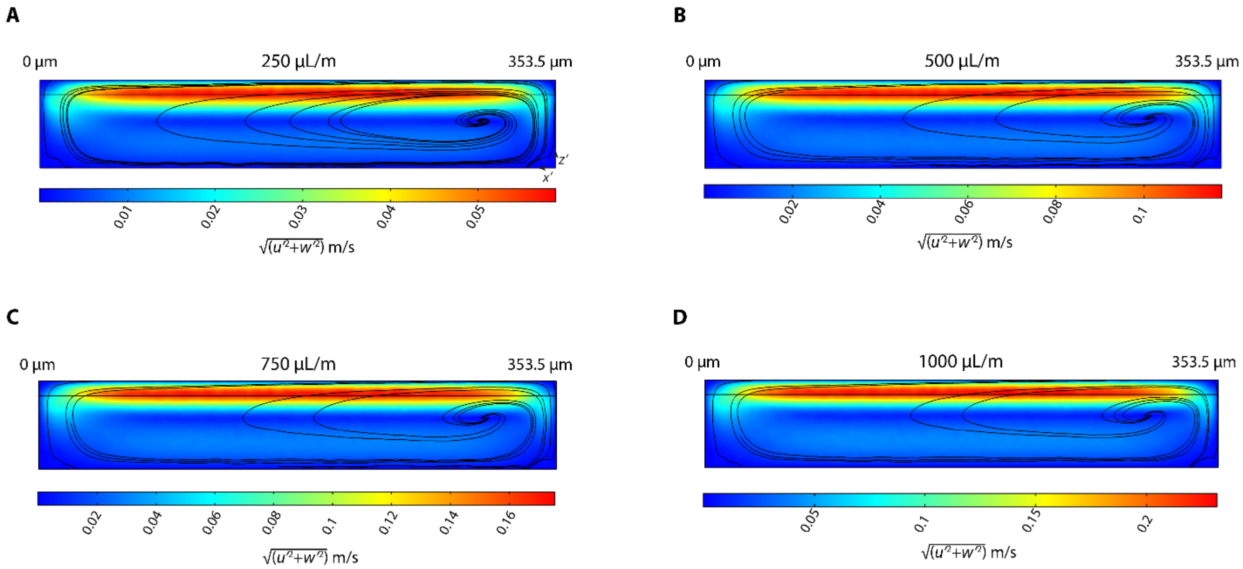

Fig. S8. Finite element simulations of fluid flow in a RAMP device show equivalent recirculation regions formed at several flow rates. Simulations were performed at flow rates of (**A**) 250 µL/min (*Re* = 25.2), (**B**) 500 µL/min (*Re* = 50.4), (**C**) 750 µL/min (*Re* = 75.6), and (**D**) 1000 µL/min (*Re* = 100.8).

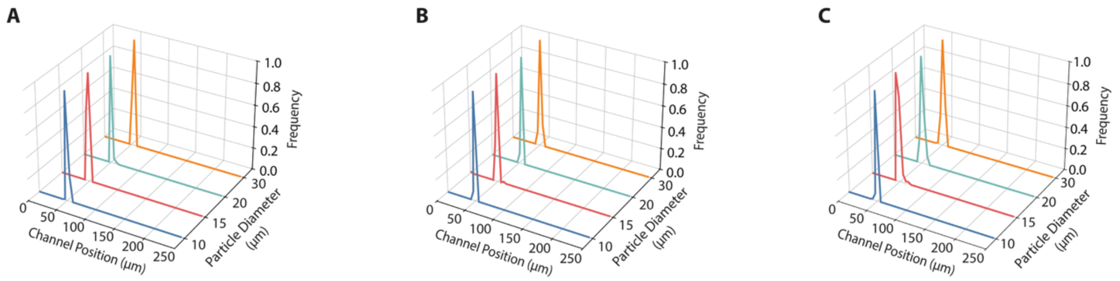

Fig. S9. RAMP aligns 10, 15, 20, and 30 µm particles across a wide range of flow rates. Histograms of particle centroid positions are shown for flow rates of (**A**) 500 µL/min, *Re* = 50.4 (*n*=118 (10 µm), 99 (15 µm), 146 (20 µm), 105 (30 µm) particles), (**B**) 750 µL/min, *Re* = 75.6 (*n*=180 (10 µm), 99 (15 µm), 71 (20 µm), 143 (30 µm) particles), and (**C**) 1000 µL/min, *Re* = 100.8 (*n*=132 (10 µm), 99 (15 µm), 165 (20 µm), 163 (30 µm) particles).

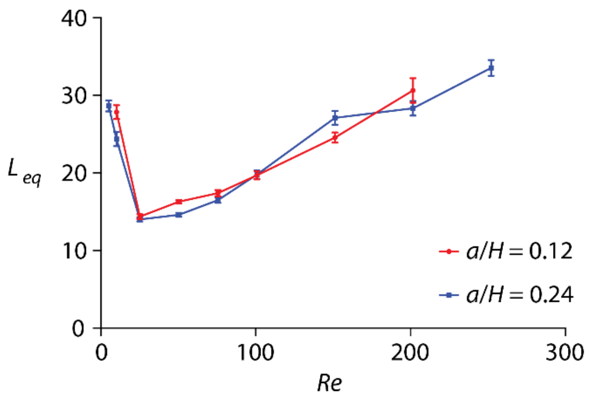

Fig. S10. Channel length required for equilibrium inertial focusing (L_{eq}) at various flow rates. At lower *Re (~5)*, inertial forces are weak, requiring a longer channel length, whereas at higher *Re*, PDMS channels inflate, reducing inertial forces and increasing L_{eq}.

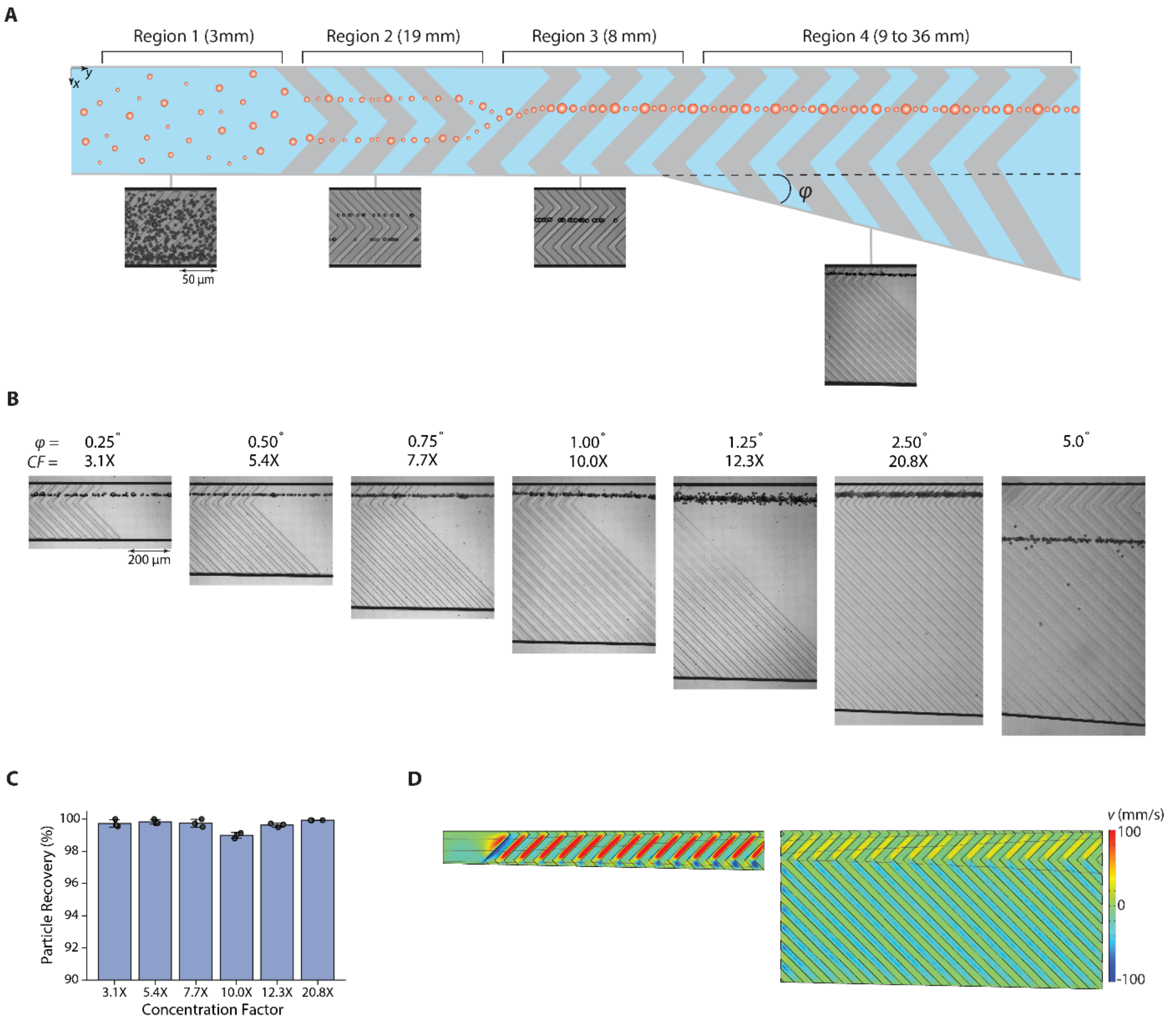

Fig. S11. RAMP siphoning device for concentration of polydisperse particles. (**A**) High-speed streak imaging showing the organization of polydisperse particles in various regions. The image in panel A, region 1, is repurposed from Figure 1. (**B**) A polydisperse mixture of particles (10 and 20 µm in diameter) was concentrated with concentration factors (CFs) of 3.1X to 20.8X. Particles in the RAMP cell concentrator become insufficiently focused at a siphoning angle of 5°, leading to diversion of the particle stream into the siphoning region. (**C**) RAMP cell concentration results in recovery greater than 99% for each CF tested (*n* = 3 replicates per CF). High-speed videos show the polydisperse particle mixture flowing through CFs of 3.1 and 12.3 (**Movie S4** and **Movie S5**). (**D**) Flow simulation results show a change in the transverse velocity profile as fluid siphoning is implemented. Gray lines show streamlines.

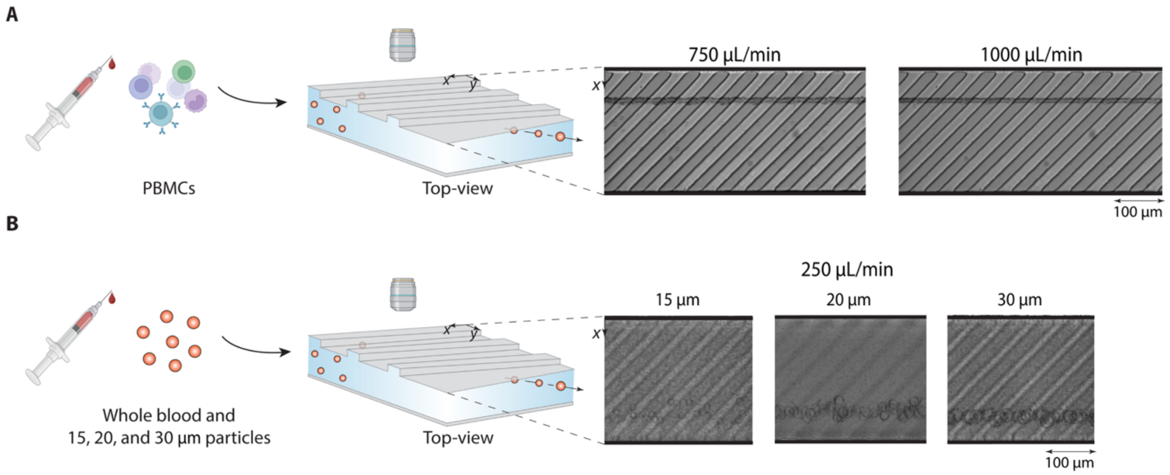

Fig. S12. Focusing of cells and particles in diverse samples. (A) Focusing peripheral blood mononuclear cells (PBMCs) using RAMP. (**B**) Focusing of 15, 20, and 30 µm particles in whole blood at 250 µL/min (*Re* = 25.2). The image of 20 µm particles was reproduced from Figure 7 for comparison with particles of multiple sizes. Created in BioRender. Payan-medina, A. (2026) https://BioRender.com/i4yx21j.

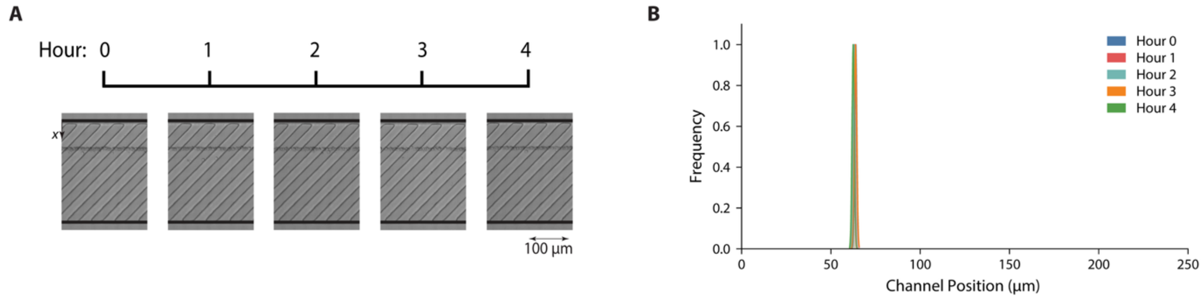

Fig. S13. Focusing of cells over a 4-hour span. (**A**) High-speed streak imaging shows that PBMCs maintain their focus position over a 4-hour span without clogging (flowing at 250 µL/min, 0.5 million cells/mL, and *Re* = 25.2). (**B**) A frequency distribution diagram of cell centroid positions at hour 0 (n = 195), 1 (n = 302), 2 (n = 105), 3 (n = 283), and 4 (n = 200).

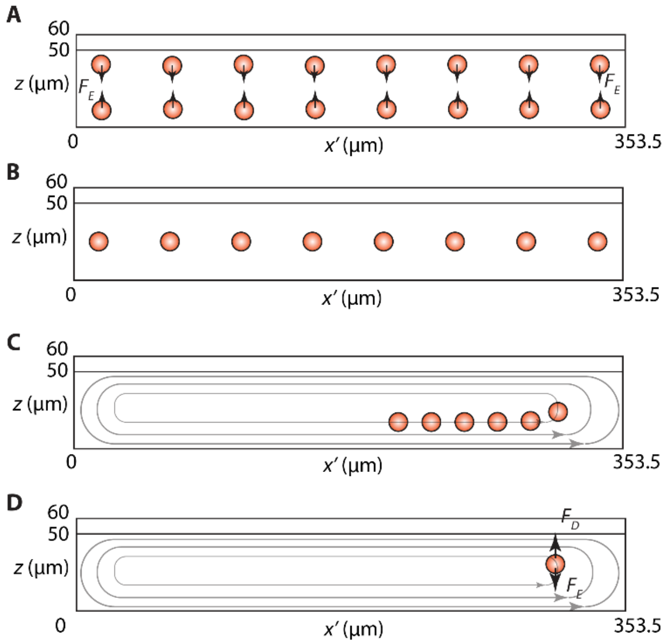

Fig. S14. Schematics of viscoelastic focusing. (**A-B**) For the conditions tested in this work, elastic forces (F_E) dominate inertial forces; as a result, particles experience an elastic force towards the center of the rectangular channel. (**C-D**) Secondary flow produced by ridges sweeps particles towards the distant sidewall of the channel, where the elastic force, directed towards the channel center, balances the drag force due to the vortex (F_D).

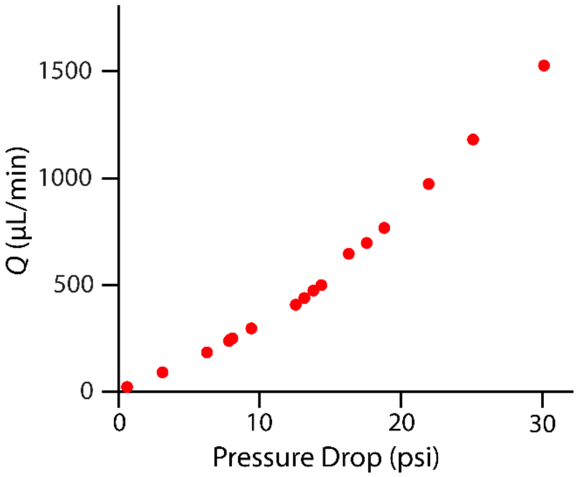

Fig. S15. Pressure drop through a 40 mm-long RAMP channel as a function of flow rate. Typically, the flow rate in a rectangular channel increases linearly with the pressure drop. However, in this case, the elastomeric PDMS channels inflate, creating a nonlinear relationship between flow rate and pressure drop.

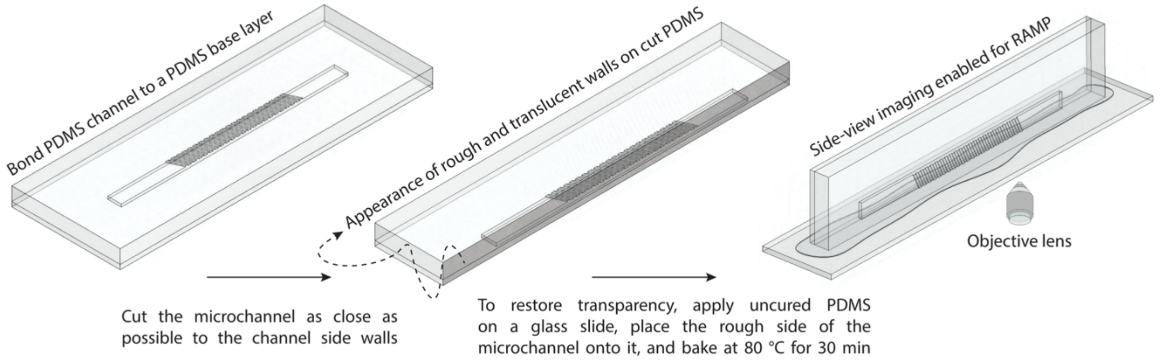

Fig. S16. Side-view imaging. Fabrication process for PDMS device with a side-view (*y*-*z* plane) window.

Table S1. An overview of microchannel geometries used for inertial focusing.

Channel Type	Channel Visual	Tunable focusing	Size-independent focusing	Flow rate-independent focusing	Focusing in height (z-direction)
Current study (RAMP)	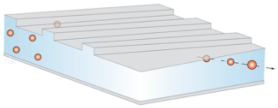	Yes	Yes	Yes	Single line
Serpentine and curvilinear	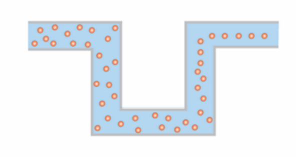	No	No	No	Double line
Obstacled curvilinear (*26*, *45*, *68–70*)	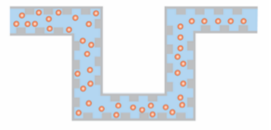	No	No	No	Double line
Asymmetric serpentine (*71*)	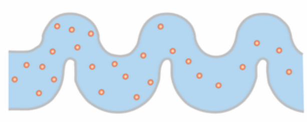	No	No	No	Double line
Spiral (*72–75*)	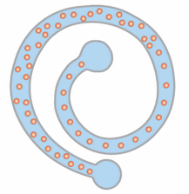	No	No	No	Double line
Obstacled-spiral (*76–78*)	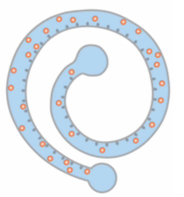	No	Yes	Yes	Double line
Contraction-expansion (*47*, *79*, *80*)	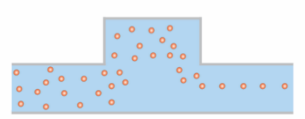	No	No	No	Double line
Reverse wavy (*46*)	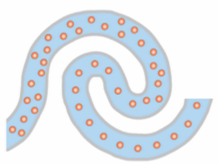	No	No	No	Double line
Straight microchannel with rectangular cross-	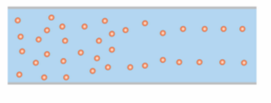	No	No	No	Double or multiple line

section (*21*, *81*)					
Straight microchannel with triangular cross-section (*82*–*84*)	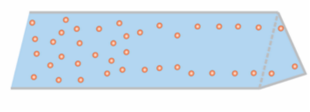	No	Yes (in some cases)	Yes (in some cases)	Single or double line
Mixed channel cross-section in a straight channel (*62*)	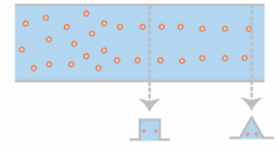	No	No	No	Single line
Stepped straight microchannel (*12*)	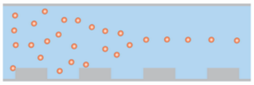	No	Yes	Yes	Single line
Grooved microchannel (*50*, *52*, *63*)	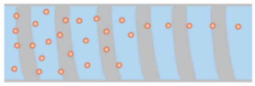	No	Yes	Yes	Single line

Table S2. Equilibrium focus positions of 10 µm particles at a flow rate of 500 µL/min (*Re*=50.4).

Central Ridge Position (µm)	Focus Position (µm)	
	10 µm Particles	10 and 15 µm Particles
15	50.8 ± 0.7	
30	67.9 ± 0.6	
60	97.5 ± 1.0	
80	109.1 ± 0.6	109.0 ± 0.6
85	113.9 ± 0.5	114.7 ± 1.1
90	119.8 ± 0.6	121.1 ± 0.6
95	126.7 ± 0.6	

Table S3. Focus positions of 10, 15, 20, and 30 μm particles at flow rates of 250 to 2500 μL/min.

Particle Size (μm)	Focus Position (μm)							
	**250 μL/min *Re* = 25.2**	**500 μL/min *Re* = 50.4**	**750 μL/min *Re* = 75.6**	**1000 μL/min *Re* = 100.8**	**1250 μL/min *Re* = 126.0**	**1500 μL/min *Re* = 151.2**	**2000 μL/min *Re* = 201.6**	**2500 μL/min *Re* = 252.0**
10	57.8 ± 0.7	55.0 ± 0.6	52.2 ± 0.5	54.0 ± 0.6	57.6 ± 1.3	58.8 ± 1.9	62.2 ± 3.0	67.2 ± 11.4
15	54.2 ± 1.3	51.1 ± 0.9	51.3 ± 5.2	51.3 ± 2.8				
20	54.4 ± 0.9	54.4 ± 1.3	56.2 ± 0.9	54.0 ± 3.2				
30	59.5 ± 2.3	56.5 ± 1.8	53.8 ± 1.8	53.1 ± 2.6				

Table S4. Focus positions of 10 µm particles at flow rates of 250 µL/min to 2500 µL/min (*Re*=25.2 to 252.0) in a rigid device.

Flow Rate (µL/min)	Mean Peak Position (µm)
250	45.4 ± 0.7
500	42.6 ± 0.5
750	42.8 ± 0.7
1000	41.4 ± 0.5
1250	42.1 ± 0.7
1500	40.9 ± 0.7
2000	47.2 ± 0.6
2500	47.0 ± 1.1

Table S5. Focusing positions of 10 µm particle in *z*-direction at flow rates ranging from 250 to 1000 µL/min captured using fluorescence microscopy in *y*-*z* plane (side-view).

Flow Rate (µL/min)	Mean Peak Position (µm)
250	30.6 ± 0.6
500	31.5 ± 0.6
750	31.5 ± 0.6
1000	32.4 ± 0.5

Table S6. Mean focus positions of 10 µm particles flowing through devices with ridge wavelengths of 30, 60, and 120 at a flow rate of 500 µL/min (*Re* = 50.4).

Ridge Wavelength	Mean Focus Position (µm)
30	46.1 ± 0.6
60	55.6 ± 0.7
120	55.8 ± 1.0

Table S7. Mean focus positions of 10 µm particles flowing through devices with ridges angled at 30°, 45°, 60°, and 70° at a flow rate of 500 µL/min (*Re* = 50.4).

Ridge Angle	Mean Focus Position (µm)
30°	54.3 ± 0.5
45°	54.6 ± 0.5
60°	53.0 ± 0.4
75°	44.3 ± 0.4

Table S8. Mean focus positions of 10 µm particles and PBMCs flowing at concentrations of 10 thousand/mL, 100 thousand/mL, 1 million/mL, and 10 million/mL at a flow rate of 500 µL/min (*Re* = 50.4).

Concentration (mL^{-1})	Mean Focus Position (µm)	
	Particles	Cells
10^4	58.3 ± 1.0	67.0 ± 2.0
10^5	57.9 ± 1.2	60.6 ± 1.8
10^6	55.5 ± 1.4	59.8 ± 2.3
10×10^6	69.7 ± 18.1	78.7 ± 18.2

Table S9. Average cell and particle recovery in RAMP concentrators (*n*=3).

Concentration Factor	Cell Recovery (%)	Particle Recovery (%)
3.1X	97.6 ± 1.1	99.7 ± 0.2
5.4X	99.5 ± 0.2	99.8 ± 0.1
7.7X	98.4 ± 0.2	99.7 ± 0.2
10.0X	95.1 ± 0.5	99.0 ± 0.2
12.3X	96.8 ± 0.8	99.6 ± 0.1
20.8X		99.9 ± 0.1

Table S10. Mean focus positions of peripheral blood mononuclear cells flowing through a RAMP device over a 4-hour time span at a flow rate of 250 µL/min (*Re* = 25.2).

Hour	Mean Focus Position (µm)
0	64.0 ± 0.6
1	63.5 ± 0.8
2	62.9 ± 0.6
3	63.8 ± 0.7
4	62.5 ± 0.6

Table S11. Deborah's number (*De*), Reynold's number (*Re*), and Elasticity number (*El*) calculations for flow rates of 50 and 100 µL/min.

Flow Rate (µL/min)	*De*	*Re*	*El*
50	130.0	1.111	117.0
100	260.0	2.222	117.0

Movie S1. Top-view of 10 µm particles flowing at 500 µL/min through a channel with central ridge structures at 80 µm from the channel sidewall (left) and 90 µm from the channel sidewall (right).

Movie S2. Top-view of 10 µm particles flowing at 500 µL/min through a channel with straight ridges.

Movie S3. Top-view of 10 (left) and 30 (right) µm particles flowing at 500 µL/min through a channel with straight ridges.

Movie S4. Top-view of 10 and 20 µm particles flowing through a RAMP concentrator with a concentration factor of 3.1 and operating pressure of 20 psi.

Movie S5. Top-view of 10 and 20 µm particles flowing through a RAMP concentrator with a concentration factor of 12.3 and operating pressure of 20 psi.

Movie S6. Top-view of peripheral blood mononuclear cells and circulating tumor cells flowing through a RAMP concentrator with a concentration factor of 3.1 and operating pressure of 15 psi.

Movie S7. Top-view of peripheral blood mononuclear cells and circulating tumor cells flowing through a RAMP concentrator with a concentration factor of 12.1 and operating pressure of 15 psi.

Movie S8. Top-view of 20 µm particles suspended in undiluted whole blood flowing in a RAMP channel at 250 µL/min.

REFERENCES

1. A. J. Mach, O. B. Adeyiga, D. Di Carlo, Microfluidic sample preparation for diagnostic cytopathology. *Lab Chip* **13**, 1011–1026 (2013).

2. J. El-Ali, P. K. Sorger, K. F. Jensen, Cells on chips. *Nature* **442**, 403–411 (2006).

3. E. Lin, L. Rivera-Báez, S. Fouladdel, H. J. Yoon, S. Guthrie, J. Wieger, Y. Deol, E. Keller, V. Sahai, D. M. Simeone, M. L. Burness, E. Azizi, M. S. Wicha, S. Nagrath, High-throughput microfluidic labyrinth for the label-free isolation of circulating tumor cells. *Cell Syst.* **5**, 295–304.e4 (2017).

4. M. Muluneh, W. Shang, D. Issadore, Track-etched magnetic micropores for immunomagnetic isolation of pathogens. *Adv. Healthc. Mater.* **3**, 1078–1085 (2014).

5. M. Toner, D. Irimia, Blood-on-a-Chip. *Annu. Rev. Biomed. Eng.* **7**, 77–103 (2005).

6. K. M. McKinnon, Flow Cytometry: An Overview. *Curr. Protoc. Immunol.* **120**, 5.1.1–5.1.11 (2018).

7. A. L. Givan, "Flow Cytometry: An Introduction" in *Flow Cytometry Protocols*, T. S. Hawley, R. G. Hawley, Eds. (Humana Press, Totowa, NJ, 2011), vol. 699 of *Methods in Molecular Biology*, pp. 1–29. https://link.springer.com/10.1007/978-1-61737-950-5_1.

8. K. Ryan, R. E. Rose, D. R. Jones, P. A. Lopez, Sheath fluid impacts the depletion of cellular metabolites in cells afflicted by sorting induced cellular stress (SICS). *Cytometry A* **99**, 921–929 (2021).

9. I. Andrä, H. Ulrich, S. Dürr, D. Soll, L. Henkel, C. Angerpointner, J. Ritter, S. Przibilla, H. Stadler, M. Effenberger, D. H. Busch, M. Schiemann, An evaluation of T-cell functionality after flow cytometry sorting revealed p38 MAPK activation. *Cytometry A* **97**, 171–183 (2020).

10. J. Oakey, R. W. Applegate, E. Arellano, D. D. Carlo, S. W. Graves, M. Toner, Particle focusing in staged inertial microfluidic devices for flow cytometry. *Anal. Chem.* **82**, 3862–3867 (2010).

11. S. C. Hur, H. T. K. Tse, D. Di Carlo, Sheathless inertial cell ordering for extreme throughput flow cytometry. *Lab Chip* **10**, 274–280 (2010).

12. A. J. Chung, D. R. Gossett, D. Di Carlo, Three dimensional, sheathless, and high-throughput microparticle inertial focusing through geometry-induced secondary flows. *Small* **9**, 685–690 (2013).

13. K. C. M. Lee, B. M. F. Chung, D. M. D. Siu, S. C. K. Ho, D. K. H. Ng, K. K. Tsia, Dispersion-free inertial focusing (DIF) for high-yield polydisperse micro-particles filtration and analysis. bioRxiv 2024.01.20.576445 (2024). https://doi.org/10.1101/2024.01.20.576445.

14. X. Wang, H. Gao, N. Dindic, N. Kaval, I. Papautsky, A low-cost, plug-and-play inertial microfluidic helical capillary device for high-throughput flow cytometry. *Biomicrofluidics* **11**, 014107 (2017).

15. A. A. S. Bhagat, S. S. Kuntaegowdanahalli, N. Kaval, C. J. Seliskar, I. Papautsky, Inertial microfluidics for sheath-less high-throughput flow cytometry. *Biomed. Microdevices* **12**, 187–195 (2010).

16. M. E. Warkiani, B. L. Khoo, L. Wu, A. K. P. Tay, A. A. S. Bhagat, J. Han, C. T. Lim, Ultra-fast, label-free isolation of circulating tumor cells from blood using spiral microfluidics. *Nat. Protoc.* **11**, 134–148 (2016).

17. J. M. Martel, M. Toner, Inertial Focusing in Microfluidics. *Annu. Rev. Biomed. Eng.* **16**, 371–396 (2014).

18. J. M. Martel, M. Toner, Particle focusing in curved microfluidic channels. *Sci. Rep.* **3**, 3340 (2013).

19. A. D. Stroock, S. K. W. Dertinger, A. Ajdari, I. Mezić, H. A. Stone, G. M. Whitesides, Chaotic mixer for microchannels. *Science* **295**, 647–651 (2002).

20. A. D. Stroock, S. K. Dertinger, G. M. Whitesides, A. Ajdari, Patterning flows using grooved surfaces. *Anal. Chem.* **74**, 5306–5312 (2002).

21. J. Zhou, I. Papautsky, Fundamentals of inertial focusing in microchannels. *Lab Chip* **13**, 1121–1132 (2013).

22. D. Di Carlo, J. F. Edd, K. J. Humphry, H. A. Stone, M. Toner, Particle segregation and dynamics in confined flows. *Phys. Rev. Lett.* **102**, 094503 (2009).

23. C. Rein, M. Toner, D. Sevenler, Rapid prototyping for high-pressure microfluidics. *Sci. Rep.* **13**, 1232 (2023).

24. E. J. Lim, T. J. Ober, J. F. Edd, S. P. Desai, D. Neal, K. W. Bong, P. S. Doyle, G. H. McKinley, M. Toner, Inertio-elastic focusing of bioparticles in microchannels at high throughput. *Nat. Commun.* **5**, 4120 (2014).

25. M. E. Warkiani, A. K. P. Tay, G. Guan, J. Han, Membrane-less microfiltration using inertial microfluidics. *Sci. Rep.* **5**, 11018 (2015).

26. Y. Liu, J. Zhang, X. Peng, S. Yan, Deciphering the evolution of inertial migration in serpentine channels. *Anal. Chem.* **96**, 14306–14314 (2024).

27. J. M. Martel, K. C. Smith, M. Dlamini, K. Pletcher, J. Yang, M. Karabacak, D. A. Haber, R. Kapur, M. Toner, Continuous flow microfluidic bioparticle concentrator. *Sci. Rep.* **5**, 11300 (2015).

28. M. Nikanjam, S. Kato, R. Kurzrock, Liquid biopsy: Current technology and clinical applications. *J. Hematol. Oncol.* **15**, 131 (2022).

29. C. S. Dai, A. Mishra, J. Edd, M. Toner, S. Maheswaran, D. A. Haber, Circulating tumor cells: Blood-based detection, molecular biology, and clinical applications. *Cancer Cell* **43**, 1399–1422 (2025).

30. A. Mishra, S.-B. Huang, T. Dubash, R. Burr, J. F. Edd, B. S. Wittner, Q. E. Cunneely, V. R. Putaturo, A. Deshpande, E. Antmen, K. A. Gopinathan, K. Otani, Y. Miyazawa, J. E. Kwak, S. Y. Guay, J. Kelly, J. Walsh, L. T. Nieman, I. Galler, P. Chan, M. S. Lawrence, R. J. Sullivan, A. Bardia, D. S. Micalizzi, L. V. Sequist, R. J. Lee, J. W. Franses, D. T. Ting, P. A. R. Brunker, S. Maheswaran, D. T. Miyamoto, D. A. Haber, M. Toner, Tumor cell-based liquid

biopsy using high-throughput microfluidic enrichment of entire leukapheresis product. *Nat. Commun.* **16**, 32 (2025).

31. N. Xiang, Z. Ni, High-throughput blood cell focusing and plasma isolation using spiral inertial microfluidic devices. *Biomed. Microdevices* **17**, 110 (2015).

32. K. Loutherback, J. D'Silva, L. Liu, A. Wu, R. H. Austin, J. C. Sturm, Deterministic separation of cancer cells from blood at 10 mL/min. *AIP Adv.* **2**, 42107 (2012).

33. C. Liu, J. Guo, F. Tian, N. Yang, F. Yan, Y. Ding, J. Wei, G. Hu, G. Nie, J. Sun, Field-free isolation of exosomes from extracellular vesicles by microfluidic viscoelastic flows. *ACS Nano* **11**, 6968–6976 (2017).

34. G. D'Avino, F. Greco, P. L. Maffettone, Particle migration due to viscoelasticity of the suspending liquid and its relevance in microfluidic devices. *Annu. Rev. Fluid Mech.* **49**, 341–360 (2017).

35. S. Yang, J. Y. Kim, S. J. Lee, S. S. Lee, J. M. Kim, Sheathless elasto-inertial particle focusing and continuous separation in a straight rectangular microchannel. *Lab Chip* **11**, 266–273 (2011).

36. R. Poole, The Deborah and Weissenberg numbers. *Rheol. Bull.* **53**, 32–39 (2012).

37. F. Del Giudice, G. Romeo, G. D'Avino, F. Greco, P. A. Netti, P. L. Maffettone, Particle alignment in a viscoelastic liquid flowing in a square-shaped microchannel. *Lab Chip* **13**, 4263–4271 (2013).

38. A. J. De Mello, N. Beard, Focus. Dealing with 'real' samples: Sample pre-treatment in microfluidic systems. *Lab Chip* **3**, 11N–20N (2003).

39. A. Mishra, T. D. Dubash, J. F. Edd, M. K. Jewett, S. G. Garre, N. M. Karabacak, D. C. Rabe, B. R. Mutlu, J. R. Walsh, R. Kapur, S. L. Stott, S. Maheswaran, D. A. Haber, M. Toner, Ultrahigh-throughput magnetic sorting of large blood volumes for epitope-agnostic isolation of circulating tumor cells. *Proc. Natl. Acad. Sci. U.S.A.* **117**, 16839–16847 (2020).

40. D. Yu, M. Humar, K. Meserve, R. C. Bailey, S. N. Chormaic, F. Vollmer, Whispering-gallery-mode sensors for biological and physical sensing. *Nat. Rev. Methods Primers* **1**, 83 (2021).

41. T. Ding, K. C. M. Lee, K. K. Tsia, T. N. Siegel, D. Di Carlo, K. Goda, Image-activated cell sorting. *Nat. Rev. Bioeng.* **3**, 890–907 (2025).

42. J. Zhu, T.-R. J. Tzeng, X. Xuan, Continuous dielectrophoretic separation of particles in a spiral microchannel. *Electrophoresis* **31**, 1382–1388 (2010).

43. S. Miltenyi, W. Müller, W. Weichel, A. Radbruch, High gradient magnetic cell separation with MACS. *Cytometry* **11**, 231–238 (1990).

44. J. Zhang, W. Li, M. Li, G. Alici, N.-T. Nguyen, Particle inertial focusing and its mechanism in a serpentine microchannel. *Microfluid. Nanofluidics* **17**, 305–316 (2014).

45. A. Özbey, M. Karimzadehkhouei, S. Akgönül, D. Gozuacik, A. Koşar, Inertial Focusing of Microparticles in Curvilinear Microchannels. *Sci. Rep.* **6**, 38809 (2016).

46. Y. Zhou, Z. Ma, Y. Ai, Sheathless inertial cell focusing and sorting with serial reverse wavy channel structures. *Microsyst Nanoeng.* **4**, 5 (2018).

47. J. Zhang, M. Li, W. H. Li, G. Alici, Inertial focusing in a straight channel with asymmetrical expansion–contraction cavity arrays using two secondary flows. *J. Micromech. Microeng.* **23**, 085023 (2013).

48. N. Venugopal Menon, S. B. Lim, C. T. Lim, Microfluidics for personalized drug screening of cancer. *Curr. Opin. Pharmacol.* **48**, 155–161 (2019).

49. J. F. Edd, A. Mishra, T. D. Dubash, S. Herrera, R. Mohammad, E. K. Williams, X. Hong, B. R. Mutlu, J. R. Walsh, F. Machado De Carvalho, B. Aldikacti, L. T. Nieman, S. L. Stott, R. Kapur, S. Maheswaran, D. A. Haber, M. Toner, Microfluidic concentration and separation of circulating tumor cell clusters from large blood volumes. *Lab Chip* **20**, 558–567 (2020).

50. Q. Zhao, J. Zhang, S. Yan, D. Yuan, H. Du, G. Alici, W. Li, High-throughput sheathless and three-dimensional microparticle focusing using a microchannel with arc-shaped groove arrays. *Sci. Rep.* **7**, 41153 (2017).

51. S. Choi, J.-K. Park, Continuous hydrophoretic separation and sizing of microparticles using slanted obstacles in a microchannel. *Lab Chip* **7**, 890–897 (2007).

52. Q. Zhao, D. Yuan, S. Yan, J. Zhang, H. Du, G. Alici, W. Li, Flow rate-insensitive microparticle separation and filtration using a microchannel with arc-shaped groove arrays. *Microfluid. Nanofluidics* **21**, 55 (2017).

53. F. E. Chrit, P. Li, T. Sulchek, A. Alexeev, Adhesion-based high-throughput label-free cell sorting using ridged microfluidic channels. *Soft Matter* **20**, 1913–1921 (2024).

54. G. Wang, K. Crawford, C. Turbyfield, W. Lam, A. Alexeev, T. Sulchek, Microfluidic cellular enrichment and separation through differences in viscoelastic deformation. *Lab Chip* **15**, 532–540 (2015).

55. S. Choi, J. M. Karp, R. Karnik, Cell sorting by deterministic cell rolling. *Lab Chip* **12**, 1427–1430 (2012).

56. A. Liu, M. Islam, N. Stone, V. Varadarajan, J. Jeong, S. Bowie, P. Qiu, E. K. Waller, A. Alexeev, T. Sulchek, Microfluidic generation of transient cell volume exchange for convectively driven intracellular delivery of large macromolecules. *Mater. Today* **21**, 703–712 (2018).

57. G. Wang, W. Mao, R. Byler, K. Patel, C. Henegar, A. Alexeev, T. Sulchek, Stiffness dependent separation of cells in a microfluidic device. *PLOS ONE* **8**, e75901 (2013).

58. M. Islam, H. Brink, S. Blanche, C. DiPrete, T. Bongiorno, N. Stone, A. Liu, A. Philip, G. Wang, W. Lam, A. Alexeev, E. K. Waller, T. Sulchek, Microfluidic sorting of cells by viability based on differences in cell stiffness. *Sci. Rep.* **7**, 1997 (2017).

59. D. Lee, S. M. Nam, J. Kim, D. Di Carlo, W. Lee, Active control of inertial focusing positions and particle separations enabled by velocity profile tuning with coflow systems. *Anal. Chem.* **90**, 2902–2911 (2018).

60. A. Bakhtiari, C. J. Kähler, Automated microparticle positioning using a pair of ultrasound-actuated microbubbles for microfluidic applications. *Microfluid. Nanofluidics* **27**, 37 (2023).

61. X. Yuan, A. Glidle, H. Furusho, H. Yin, A 3D hydrodynamic flow-focusing device for cell sorting. *Microfluid. Nanofluidics* **25**, 23 (2021).

62. J. Kim, J. Lee, C. Wu, S. Nam, D. Di Carlo, W. Lee, Inertial focusing in non-rectangular cross-section microchannels and manipulation of accessible focusing positions. *Lab Chip* **16**, 992–1001 (2016).

63. Q. Zhao, D. Yuan, S.-Y. Tang, G. Yun, S. Yan, J. Zhang, W. Li, Top sheath flow-assisted secondary flow particle manipulation in microchannels with the slanted groove structure. *Microfluid. Nanofluidics* **23**, 6 (2019).

64. K. C. M. Lee, M. Wang, K. S. E. Cheah, G. C. F. Chan, H. K. H. So, K. K. Y. Wong, K. K. Tsia, Quantitative phase imaging flow cytometry for ultra-large-scale single-cell biophysical phenotyping. *Cytometry A* **95**, 510–520 (2019).

65. D. Yuan, J. Zhang, R. Sluyter, Q. Zhao, S. Yan, G. Alici, W. Li, Continuous plasma extraction under viscoelastic fluid in a straight channel with asymmetrical expansion–contraction cavity arrays. *Lab Chip* **16**, 3919–3928 (2016).

66. X. Lu, X. Xuan, Continuous microfluidic particle separation via elasto-inertial pinched flow fractionation. *Anal. Chem.* **87**, 6389–6396 (2015).

67. N. V. Jordan, A. Bardia, B. S. Wittner, C. Benes, M. Ligorio, Y. Zheng, M. Yu, T. K. Sundaresan, J. A. Licausi, R. Desai, R. M. O'Keefe, R. Y. Ebright, M. Boukhali, S. Sil, M. L. Onozato, A. J. Iafrate, R. Kapur, D. Sgroi, D. T. Ting, M. Toner, S. Ramaswamy, W. Haas, S. Maheswaran, D. A. Haber, HER2 expression identifies dynamic functional states within circulating breast cancer cells. *Nature* **537**, 102–106 (2016).

68. H. Cha, H. Fallahi, Y. Dai, S. Yadav, S. Hettiarachchi, A. McNamee, H. An, N. Xiang, N.-T. Nguyen, J. Zhang, Tuning particle inertial separation in sinusoidal channels by embedding periodic obstacle microstructures. *Lab Chip* **22**, 2789–2800 (2022).

69. H. Cha, H. A. Amiri, S. Moshafi, A. Karimi, A. Nikkhah, X. Chen, H. T. Ta, N.-T. Nguyen, J. Zhang, Effects of obstacles on inertial focusing and separation in sinusoidal channels: An experimental and numerical study. *Chem. Eng. Sci.* **276**, 118826 (2023).

70. J. Zhang, S. Yan, R. Sluyter, W. Li, G. Alici, N.-T. Nguyen, Inertial particle separation by differential equilibrium positions in a symmetrical serpentine micro-channel. *Sci. Rep.* **4**, 4527 (2014).

71. D. Di Carlo, D. Irimia, R. G. Tompkins, M. Toner, Continuous inertial focusing, ordering, and separation of particles in microchannels. *Proc. Natl. Acad. Sci. U.S.A.* **104**, 18892–18897 (2007).

72. M. E. Warkiani, G. Guan, K. B. Luan, W. C. Lee, A. A. S. Bhagat, P. Kant Chaudhuri, D. S.-W. Tan, W. T. Lim, S. C. Lee, P. C. Y. Chen, C. T. Lim, J. Han, Slanted spiral microfluidics for the ultra-fast, label-free isolation of circulating tumor cells. *Lab Chip* **14**, 128–137 (2014).

73. S. S. Kuntaegowdanahalli, A. A. S. Bhagat, G. Kumar, I. Papautsky, Inertial microfluidics for continuous particle separation in spiral microchannels. *Lab Chip* **9**, 2973–2980 (2009).

74. N. Herrmann, P. Neubauer, M. Birkholz, Spiral microfluidic devices for cell separation and sorting in bioprocesses. *Biomicrofluidics* **13**, 061501 (2019).

75. A. A. S. Bhagat, S. S. Kuntaegowdanahalli, I. Papautsky, Continuous particle separation in spiral microchannels using dean flows and differential migration. *Lab Chip* **8**, 1906–1914 (2008).

76. J. Guo, S. Shen, N. Zhang, F. Zhang, Y. Niu, Y. Wu, Secondary flow enhancement in ultra-low aspect ratio microchannels with ordered Micro-obstacles for high-throughput cell focusing. *Microchem. J.* **216**, 114570 (2025).

77. S. Shen, Y. Zhang, K. Yang, H. Chan, W. Li, X. Li, C. Tian, Y. Niu, Flow-rate-insensitive plasma extraction by the stabilization and acceleration of secondary flow in the ultralow aspect ratio spiral channel. *Anal. Chem.* **95**, 18278–18286 (2023).

78. L. Zhao, M. Gao, Y. Niu, J. Wang, S. Shen, Flow-rate and particle-size insensitive inertial focusing in dimension-confined ultra-low aspect ratio spiral microchannel. *Sens. Actuators B* **369**, 132284 (2022).

79. G.-Y. Kim, J. Son, J.-I. Han, J.-K. Park, Inertial microfluidics-based separation of microalgae using a contraction–expansion array microchannel. *Micromachines* **12**, 97 (2021).

80. M. G. Lee, S. Choi, J.-K. Park, Inertial separation in a contraction–expansion array microchannel. *J. Chromatogr. A* **1218**, 4138–4143 (2011).

81. C. Liu, G. Hu, X. Jiang, J. Sun, Inertial focusing of spherical particles in rectangular microchannels over a wide range of Reynolds numbers. *Lab Chip* **15**, 1168–1177 (2015).

82. P. Mukherjee, X. Wang, J. Zhou, I. Papautsky, Single stream inertial focusing in low aspect-ratio triangular microchannels. *Lab Chip* **19**, 147–157 (2019).

83. E. Cho, J. Kim, M. K. Aslan, Y. Meng, S. Stavrakis, A. deMello, Tunable viscoelastic size-based particle separation in straight microchannels with triangular cross-sections. *Sens. Actuators B* **414**, 135892 (2024).

84. J. Kim, J.-R. Lee, T.-J. Je, E. Jeon, W. Lee, Size-Dependent Inertial Focusing Position Shift and Particle Separations in Triangular Microchannels. *Anal. Chem.* **90**, 1827–1835 (2018).
